# CSMET: Comparative Genomic Motif Detection via Multi-Resolution Phylogenetic Shadowing

**DOI:** 10.1371/journal.pcbi.1000090

**Published:** 2008-06-06

**Authors:** Pradipta Ray, Suyash Shringarpure, Mladen Kolar, Eric P. Xing

**Affiliations:** School of Computer Science, Carnegie Mellon University, Pittsburgh, Pennsylvania, United States of America; Duke University, United States of America

## Abstract

Functional turnover of transcription factor binding sites (TFBSs), such as whole-motif loss or gain, are common events during genome evolution. Conventional probabilistic phylogenetic shadowing methods model the evolution of genomes only at nucleotide level, and lack the ability to capture the evolutionary dynamics of functional turnover of aligned sequence entities. As a result, comparative genomic search of non-conserved motifs across evolutionarily related taxa remains a difficult challenge, especially in higher eukaryotes, where the *cis*-regulatory regions containing motifs can be long and divergent; existing methods rely heavily on specialized pattern-driven heuristic search or sampling algorithms, which can be difficult to generalize and hard to interpret based on phylogenetic principles. We propose a new method: Conditional Shadowing via Multi-resolution Evolutionary Trees, or CSMET, which uses a context-dependent probabilistic graphical model that allows aligned sites from different taxa in a multiple alignment to be modeled by either a background or an appropriate motif phylogeny conditioning on the functional specifications of each taxon. The functional specifications themselves are the output of a phylogeny which models the evolution not of individual nucleotides, but of the overall functionality (e.g., functional retention or loss) of the aligned sequence segments over lineages. Combining this method with a hidden Markov model that autocorrelates evolutionary rates on successive sites in the genome, CSMET offers a principled way to take into consideration lineage-specific evolution of TFBSs during motif detection, and a readily computable analytical form of the posterior distribution of motifs under TFBS turnover. On both simulated and real *Drosophila cis*-regulatory modules, CSMET outperforms other state-of-the-art comparative genomic motif finders.

## Author Summary

Functional turnover of transcription factor binding sites (TFBSs), such as whole-motif loss or gain, are common events during genome evolution, and play a major role in shaping the genome and regulatory circuitry of contemporary species. Conventional methods for searching non-conserved motifs across evolutionarily related species have little or no probabilistic machinery to explicitly model this important evolutionary process; therefore, they offer little insight into the mechanism and dynamics of TFBS turnover and have limited power in finding motif patterns shaped by such processes. In this paper, we propose a new method: Conditional Shadowing via Multi-resolution Evolutionary Trees, or CSMET, which uses a mathematically elegant and computationally efficient way to model biological sequence evolution at both nucleotide level at each individual site, and functional level of a whole TFBS. CSMET offers the first principled way to take into consideration lineage-specific evolution of TFBSs and CRMs during motif detection, and offers a readily computable analytical form of the posterior distribution of motifs under TFBS turnover. Its performance improves upon current state-of-the-art programs. It represents an initial foray into the problem of statistical inference of functional evolution of TFBS, and offers a well-founded mathematical basis for the development of more realistic and informative models.

## Introduction

Phylogenetic shadowing techniques based on probabilistic molecular evolution models have been widely used in various comparative genomic analyses to uncover sequence entities believed to be conserved across species [1]–[4]. It is nothworthy that in the literature, the term “Phylogenetic Shadowing” has sometimes been (unnecessarily) narrowed down to refer to methods tailored specifically to the case of analyzing extremely closely related species, after its successful application to functional annotation of the primate genomes [1]. Here we adopt a more general interpretation reflecting the long-standing evolutionary principles and inferential technique underlying such analysis, rather than the choice of the study subjects. It refers to the class of methods that treat evolutionarily related entities as outcomes of some stochastic processes structured as a phylogeny, whereby the relationships between the studied entities can be inferred and utilized to unravel their underlying characteristics of interest. Typically, extant phylogenetic shadowing methods employ either a nucleotide (nt) or an amino-acid (aa) substitution process to model the evolution of orthologous entities, such as genes or proteins of interest at every nt or aa site. There are two key assumptions underlying the basic form of these approaches. 1) The orthology of the sequence entities across taxa, as captured by a multiple sequence alignment, is *complete* in the sense that there is no functional turnover of the aligned entities (e.g., no loss or gain of a gene) in any of the taxa; so that all aligned sequences can be modeled as descendants of a common ancestor following a *single* evolutionary tree model unique to the function (e.g., either gene or background) of the sequence entities. 2) Every site in the same entity evolves independently. Although not realistic, such a *complete* and *independent* shadowing model can lead to efficient algorithms for scoring aligned sequences; and in practice it works well for modeling large and highly conserved functional entities such as gene coding regions in phylogenetically closely related taxa, and it has led to a number of successful comparative genomic gene finders [5]–[7].

**Figure 1:**
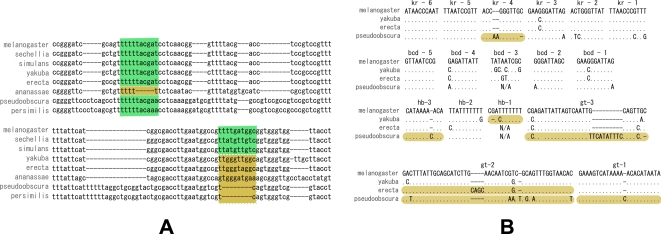
A demonstration of motif turnover. (A) Two examples of multiple alignments of *Drosophila* CRMs, showing functional turnover in known TFBSs. The first one (top) shows an instance of binding site loss in *D. ananassae*, the motif in question being *Caudal*, in the *Hairy 6* CRM. The second one (bottom) shows more instances of TFBS loss/gain. This example depicts a turnover with only *melanogaster*, *simulans,* and *sechellia* retaining the binding site functionality. (B) Putative TFBSs in *eve*2 enhancer across 4 taxa: *D. melanogaster*, *D. yakuba*, *D. erecta* and *D. pseudoobscura*. (Extracted and modified from Figure 4 in [11].) Notice that orthologs of *melanogaster* motifs bcd-3 and hb can not be identified from some of the other taxa.

**Figure 2:**
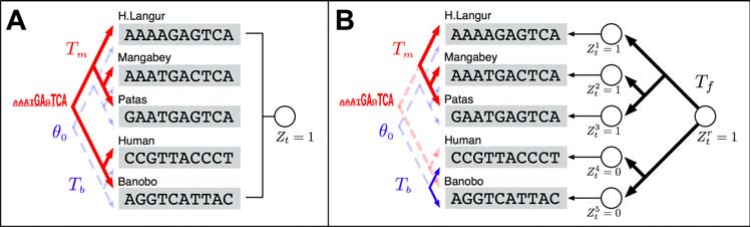
Diagrams showing the underlying generative models underlying basic phylogenetic shadowing approaches and the CSMET approach. (A) The basic mixture of full-phylogeny model underlying PhyloHMM and EMnEM, where functional homogeneity across aligned sequences is assumed, and all aligned taxa (i.e., rows) are either under a full motif phylogeny (when *Z_t_* = 1) or a full background phylogeny (when *Z_t_* = 0). (B) The conditional shadowing model underlying CSMET, with an explicit evolutionary model *T_f_* for species-specific functional turnover, and partial motif or background phylogenies over subsets of taxa according to the turnover status. See the Results section for explanations of the notations.

Unlike genes, where functional turnover usually occurs only in distant species and the complete orthology assumption is largely satisfied when sequences are aligned across phylogenetically closely related species, short and degenerate sequence patterns such as transcription factor (TF) binding sites (i.e., motifs) exhibit frequent turnover even across closely related taxa, such as various fruit fly species [8] (Figure 1). As we will discuss shortly, the functional heterogeneity of aligned regions across different taxa due to motif turnover often renders the conventional phylogenetic shadowing models inappropriate for comparative genomic motif finding. Some recent methods combine scoring functions modified from classic molecular evolution models with more flexible heuristic partial alignment search, and exhibit better sensitivity to non-conserved motifs [9],[10], but they offer little insight into the evolutionary dynamics of motif turnover and can have substantial computational complexity. In this paper, we present a principled approach that addresses the “incomplete orthology” issue arising from either functional gain/loss such as motif turnover or imperfect sequence alignment. We propose a new algorithm for searching binding sites of given TFs in multiple genomes based on a novel multi-resolution evolutionary model named CSMET. CSMET stands for Conditional Shadowing via Multi-resolution Evolutionary Trees. It explicitly models motif turnover across species through a “low resolution” phylogeny defined by a *functional substitution process*. Conditioning on the motif turnover states, which specify the presence or absence of TFBS functionality in each taxon, at any given location, specific “high resolution” phylogenies defined by function-specific *nucleotide substitution processes* are applied to different subsets (corresponding to taxa with different turnover status) of the aligned sequences at the attendant location. The model thereby captures function-specific sequence evolution in every taxon rather than subjecting all taxa to the same phylogeny as in the conventional model (Figure 2).

### Comparative Genomic Motif Search Under Incomplete Orthology

We concern ourselves with uncovering motifs in eukaryotic *cis*-regulatory modules (CRM) from multiple evolutionarily related species, such as the members from the *Drosophila* clade. Due to high degeneracy of motif instances, and complex motif organization within the CRMs, pattern-matching-based motif search in higher eukaryotes remains a difficult problem, even when representations such as the position weight matrices (PWMs) of the motifs are given. Extant methods that operate on a single genome or simpler organisms such as yeast often yield a large number of false positives, especially when the sequence to be examined spans a long region (e.g., tens of thousands of bps) beyond the basal promoters, where possible CRMs could be located. As in gene finding, having orthologous sequences from multiple evolutionarily related taxa can potentially benefit motif detection because a reasonable alignment of these sequences could enhance the contrast of sequence conservation in motifs with respect to that of the non-motif regions, However, the alignment quality of non-coding regions is usually significantly worse than that of the coding regions, so that the aligned motif sequences are not reliably orthologous. This is often unavoidable even for the best possible local alignment software because of the short lengths and weak conservation of TFBSs. When applying a standard shadowing model on such alignments, motif instances aligned with non-orthologous sequences or gaps can be hard to identify due to low overall shadowing score of the aligned sequences (Figure 1A).

In addition to the *incomplete orthology* due to imperfect alignment, a more serious concern comes from a legitimate uncertainty over the actual functional orthology of regions that are *alignment-wise* orthologous.

A number of recent investigations have shown that TFBS loss and gain are fairly common events during genome evolution [8],[12]. For example, Patel et al [13] showed that aligned “motif sites” in orthologous CRMs in the *Drosophila* clade may have varying functionality in different taxa. Such cases usually occur in regions with reduced evolutionary constraints, such as regions where motifs are abundant, or near a duplication event. The sequence dissimilarities of CRMs across taxa include indel events in the spacers, as well as gains and losses of binding sites for TFs such as the bcd-3 and hb-1 motifs in the *evenskipped* stripe 2 (*eve2*) (Figure 1B). A recent statistical analysis of the *Zeste* binding sites in several *Drosophila* taxa also revealed existence of large-scale functional turnover [12]. Nevertheless, the fact that sequence similarity is absent does not necessarily mean that the overall functional effect of the CRM as a whole is vastly different. In fact, for the *Drosophila* clade, despite the substantial sequence dissimilarity in gap-gene CRMs such as *eve2*, the expression of these gap genes shows similar spatio-temporal stripe patterns across the taxa [8],[13].

Although a clear understanding of the evolutionary dynamics underlying such inter- and intra-taxa diversity is still lacking, it is hypothesized that regulatory sequences such as TFBSs and CRMs may undergo adaptive evolution via stabilizing selections acting synergistically on different loci within the sequence elements [8],[12], which causes site evolution to be non-*iid* and non-*isotropic* across all taxa. In such a scenario, it is crucial to be able to model the evolution of biological entities not only at the resolution of individual nucleotides, but also at more macroscopic levels, such as the functionality of whole sequence elements such as TFBSs over lineages. To our knowledge, so far there have been few attempts along this line, especially in the context of motif detection. The CSMET model presented in this paper intends to address this issue.

### Related Work

Orthology-based motif detection methods developed so far are mainly based on nucleotide-level conservation. Some of the methods do not resort to a formal evolutionary model [14], but are guided by either empirical conservation measures [15]–[17], such as parsimonious substitution events or window-based nucleotide identity, or by empirical likelihood functions not explicitly modeling sequence evolution [4],[18],[19]. The advantage of these non-phylogeny based methods lies in the simplicity of their design, and their non-reliance on strong evolutionary assumptions. However, since they do not correspond to explicit evolutionary models, their utility is restricted to purely pattern search, and not for analytical tasks such as ancestral inference or evolutionary parameter estimation. Some of these methods employ specialized heuristic search algorithms that are difficult to scale up to multiple species, or generalize to aligned sequences with high divergence.

Phylogenetic methods such as EMnEM [20], MONKEY [21], and our in-house implementation of PhyloHMM (originally implemented in [1] for gene finding, but in our own version tailored for motif search) explicitly adopt a *complete* and *independent* shadowing model at the nucleotide level. These methods are all based on the assumption of homogeneity of functionality across orthologous nucleotides, which is not always true even among relatively closely related species (e.g., of divergence less than 50 mya in *Drosophila*).

Empirical estimation and simulation of turnover events is an emerging subject in the literature [12],[22], but to our knowledge, no explicit evolutionary model for functional turnover has been proposed and brought to bear in comparative genomic search of non-conserved motifs. Thus our CSMET model represents an initial foray in this direction. Closely related to our work, two recent algorithms, rMonkey [12]—an extension over the MONKEY program, and PhyloGibbs [9]—a Gibbs sampling based motif detection algorithm, can also explicitly account for differential functionality among orthologs, both using the technique of shuffling or reducing the input alignment to create well conserved local subalignments. But in both methods, no explicit functional turnover model has been used to infer the turnover events. Another recent program, PhyME [10], partially addresses the incomplete orthology issue via a heuristic that allows motifs only present in a pre-chosen *reference* taxon to be also detectable, but it is not clear how to generalize this ability to motifs present in arbitrary combination of other taxa, and so far no well-founded evolutionary hypothesis and model is provided to explain the heuristic. Non-homogeneous conservation due to selection across aligned sites has also been studied in DLESS [23] and PhastCons [24], but unlike in CSMET, no explicit substitution model for lineage-specific functional evolution was used in these algorithms, and the HMM-based model employed there makes it computationally much more expensive than CSMET to systematically explore all possible evolutionary hypotheses. A notable work in the context of protein classification proposed a phylogenomic model over protein functions, which employs a regression-like functional to model the evolution of protein functions represented as feature vectors along lineages in a *complete* phylogeny [25], but such ideas have not been explored so far for comparative genomic motif search.

Various nucleotide substitution models, including the Jukes-Cantor 69 (JC69) model [26], and the Felsenstein 81 (F81) model [27], have been employed in current phylogenetic shadowing or footprinting algorithms. PhyloGibbs and PhyME use an analogue of F81 proposed in [28], which is one of the simplest models to handle arbitrary stationary distributions, necessary to model various specific PWMs of motifs. Both PhyME and PhyloGibbs also offer an alternative to use a simplified star-phylogeny to replace the phylogenetic tree when dealing with a large number of taxa, which corresponds to an even simpler substitution process.

### The CSMET Approach

Our CSMET model differs from these existing methods in several important ways. First, it uses a different evolutionary model based on a coupled-set of both functional and nucleotide substitution processes, rather than a single nucleotide substitution model to score every alignment block. Second, it uses a more sophisticated and popular nucleotide substitution process based on the Felsenstein84 (F84) model [29], which captures the transition/transversion bias. Third, it employs a hidden Markov model that explicitly models autocorrelation of evolutionary rates on successive sites in the genome. Fourth, it uses an efficient deterministic inference algorithm that is linear to the length of the input sequence and either exponential (under a full functional phylogeny) or linear (under a star-shaped functional phylogeny) to the number of the aligned taxa, rather than the Monte Carlo or heuristic search algorithms that require long convergence times.

Essentially, CSMET is a context-dependent probabilistic graphical model that allows a single column in a multiple alignment to be modeled by multiple evolutionary trees conditioned on the functional specifications of each row (i.e., the functional identity of a substring in the corresponding taxon) (Figure 2). When conjoined with a hidden Markov model that auto-correlates the choices of different evolutionary rates on the phylogenetic trees at different sites, we have a stochastic generative model of phylogenetically related CRM sequences that allows both binding site turnover in arbitrary subsets of taxa, and coupling of evolutionary forces at different sites based on the motif organizations within CRMs. Overall, CSMET offers an elegant and efficient way to take into consideration complex evolutionary mechanisms of regulatory sequences during motif detection. When such a model is properly trained on annotated sequences, it can be used for comparative genomic motif search in all aligned taxa based on a posterior probabilistic inference algorithm. This model can be also used for *de novo* motif finding as programs such as PhyloGibbs and PhyME, with a straightforward extension of the inference procedure that couples the training and prediction routines in an expectation-maximization (EM) iteration on unannotated sequence alignments. In this paper, we focus on supervised motif search in higher eukaryotic genomes.

We compare CSMET with representative competing algorithms, including EMnEm, PhyloHMM, PhyloGibbs, and a mono-genomic baseline Stubb (which uses an HMM on single species) on both simulated data, and a pre-aligned Drosophila dataset containing 14 developmental CRMs for 11 aligned *Drosophila* species. Annotations for motif occurrences in *D. melanogaster* of 5 gap-gene TFs - *Bicoid*, *Caudal*, *Hunchback*, *Kruppel* and *Knirps* - were obtained from the literature. We show that CSMET outperforms the other methods on both synthetic and real data, and identifies a number of previously unknown occurrences of motifs within and near the study CRMs. The CSMET program, the data used in this analysis, and the predicted TFBS in *Drosophila* sequences, are available for download at http://www.sailing.cs.cmu.edu/csmet/.

## Results

### The CSMET Model

#### Model for phylogenetically related motif sequences

To motivate and explain the statistical foundation and biological rationale underlying the CSMET model, we begin with a brief description of a conventional model for phylogenetically related sequences based on the classical molecular substitution process, where functional turnover of motifs is not explicitly modeled. This model will be used as a component in our proposed model.

Consider a multiple alignment of *M* instances of a motif of length *L*. Let **A** denote an *M* × *L* matrix containing *M* rows *a*_1_,…,*a_M_*, each representing an instance of this motif, i.e., *a_i_* ≡ [*a_i_*_,1_,…,*a_i,L_*], where 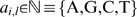. Due to the stochastic nature of the sequence composition of TFBSs, a popular representation of a motif pattern is the *position weight matrix* (PWM), **θ**≡(*θ*_1_,…,*θ_L_*), of which each column vector *θ_l_* defines a *multinomial* probability distribution of the nucleotides observed at the *l^th^* position of instances of this motif. That is, 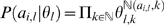, where 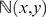 is an indicator function that equals to 1 when *x* = *y* and 0 otherwise. Under a PWM, all sites in the motif are assumed to be mutually independent, thus the probability of a length-*L* instance is simply a product of the probabilities of nucleotides at every site: 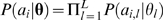. When the motif instances in **A** are from different genomic locations of a single species (i.e., they are phylogenetically *unrelated*), the likelihood of the aligned motifs **A** is simply a product of the likelihoods of every instance *a_i_*, 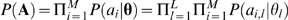, which means all the rows in **A** are independent of each other (although in reality, they might not evolve independently.)

If **A** contains *M* phylogenetically related motif instances each from a different species, then a straightforward way to model the likelihood of **A** is to assume that the instances therein from different taxa are *shadowed* by a phylogenetic tree that defines a nucleotide-level substitution process from an ancestral sequence [29],[30] (Figure 2A). Our proposed method uses this model as a building block.

Formally, a phylogenetic shadowing model *T_m_* for a motif is a *tree-likelihood model* specified by a four-tuple **{θ**,**τ**,**β**,**λ}**, where **θ**≡(*θ*_1_,…*θ_L_*) represents the equilibrium nucleotide distributions at the root of the evolutionary tree of every site within the motif; **τ**≡(*τ*_1_,…,*τ_L_*) denotes the (usually identical) topologies of the evolutionary trees of every site; **β**≡(*β*_1_,…,*β_L_*) denotes the sets of branch lengths of the evolutionary trees; and **λ** represents where necessary some additional evolutionary parameters of the motif depending on the specific nucleotide substitution models. Under a phylogenetic shadowing model, the probability distribution of nucleotides in any taxon that corresponds to a leaf conditioning on its predecessor in the tree can be derived based on a continuous-time Markov model of nucleotide substitution along the tree branches [30]. We employ the F84 substitution model parameterized by a given equilibrium distribution, a transition/tranversion ratio *ρ*, and a total substitution rate *μ* that can be estimated from training data [29]. Detailed derivation and explicit expressions are provided in Materials and Methods.

Typically, we can use the PWM of the motif as the equilibrium distribution of the motif phylogeny. For simplicity, one can also assume that all sites within the motif share the same topology *τ* and the same branch lengths *β*. This means that the evolutionary processes underlying each site within the motif are homogeneous. Similarly, we can define *Tb* ≡ {*θ_b_, τ_b_, β_b_, λ_b_*} for the background. Assuming that sites within the motif evolve independently, the likelihood of *M* aligned *L*-mers can be expressed as:
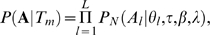
(1)where *A_l_* denotes the *l^th^* column in **A**, and 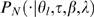 is the marginal likelihood of the leaves under an motif-site-specific evolutionary tree 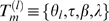 for nucleotide substitution, which can be computed using Felsenstein's pruning algorithm [30], as detailed in Materials and Methods.

To model a multiple alignment of regulatory regions that is *N* base-pairs long and contains motifs at unknown positions, we can assume that every *L*-mer block in the alignment can correspond to either a motif sequence, or the background, specified by a hidden *functional state Z_t_*, where *t* denotes the position of the left-most column of the block in the alignment. (For simplicity, we consider only one motif type here, but the formulation readily generalizes to multiple motif types.) The state sequence **Z** ≡ *Z*_1:*N*_ can be thought of as a functional annotation sequence of an ancestral regulatory region of length *N*. In the EMnEM model [20], the *Z_t_*'s are assumed to be independently sampled from a Binomial distribution of motif and background states, similar to the classic mixture models of motif underlying MEME (Figure 2A). In a PhyloHMM originally proposed in [3] for comparative gene finding, which can be easily extended for motif search, *Z*_1:*N*_ can follow a hidden Markov model that captures the transition probabilities between background and motifs.

#### Model for motif turnover

A caveat of the phylogenetic shadowing model described above is that, at every location *t*, the functionality indicator *Z_t_* must apply to all the taxa (i.e., rows) in the alignment (as illustrated in Figure 2A), meaning that the aligned substrings from all taxa at this position are derived from the same evolutionary tree (either the motif or the background tree, depending on the value of *Z_t_*; when *Z_t_* is hidden, this results in a mixture of two complete trees). This is a strong orthologous assumption which insists that every row in the alignment block must have evolved from the same most recent common ancestor (MRCA) according to the same molecular evolution model. This assumption might not be valid for every region in the alignment due to abrupt functional turnover such as whole motif insertion/deletion, or due to imperfect alignment that fails to identify the true sequence orthology.

We assume that every sequence segment in an alignment block, generically referred as **A** *_t_* where *t* denotes the left-most position of the alignment, has its own functionality indicator 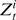. Generalizing the molecular evolution model for base substitution, we posit that the functional annotation vector 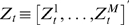 of a block of aligned segments are themselves governed by a *coarser-grained evolutionary tree* that models the evolution of the functionalities of the attendant segments in different taxa (Figure 2B). We refer to this evolutionary tree as a (functional) *annotation tree* (or, interchangeably, a functional phylogeny), denoted by *T_f_* ≡ {*α*, *τ_f_*, *b_f_, λ_f_*}. In such a tree model, each leaf represents a random variable 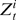 whose value reveals the functional status (i.e., being a motif, background, or more detailed function information such as motif types, etc.) of the segment from taxon *i*, and the root is characterized by a hypothetical ancestral functionality indicator 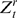 and an equilibrium distribution *α*. Along the branches of this tree, the functional states evolve according to a *functionality substitution* model, in much the same way the nucleotides do under a *molecular substitution* model, except that now the model parameters *T_f_* are fitted differently (we will return to this point in the Materials and Methods section) and the evolutionary dynamics can also have richer structures. For example, in the model proposed by [25] for protein function evolution, the evolutionary dynamics were captured by a logistic regression rather than a constant-rate continuous-time Markov process used in standard molecular substitution models. For simplicity,here we adopt a simple JC69 model for functionality substitution, which is denoted as *P_F_*(*Z_t_*|*T_f_*) (see Equation 4 in Materials and Methods), and defer the exploration of richer models to future research. In summary, the functional phylogeny *T_f_* models the quantum changes of functional elements (rather than the fine-grained changes at the nucleotide level) during evolution in terms of whether an entire functional element is preserved, lost, or emerged, during the course of speciation.

#### Conditional shadowing under motif turnover

To capture the effect of motif turnover, we assume that *conditioning* on the functional states of all rows (i.e., species), which are represented as a random column vector 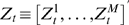 distributed according to the functional phylogeny specified by *T_f_*, the sequences in alignment block *t* admit either a *marginal* motif phylogeny or a *marginal* background phylogeny. As shown in Figure 2B, typically, for a given block, only a subset of the rows 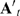 correspond to conserved instances of a motif (e.g., rows 1, 2, and 3), and therefore their joint probability is defined by a *marginal phylogeny* 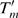 of the full motif phylogeny (i.e., the subtree highlighted by solid red lines in Figure 2B). The remaining part of the motif phylogeny (represented by the subtree in dotted red lines in Figure 2B), which corresponds to taxa where the corresponding motifs had turned-over to background sequences, needs to be marginalized out. We can efficiently compute the likelihood of the preserved motif instances 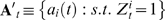 under the marginal motif phylogeny 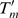, expressed as 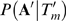 using the standard pruning algorithm. Similarly, the subset of rows 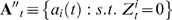 corresponding to the background or merely gaps admit a *marginal background phylogeny* 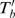 (e.g., the blue tree with leaves only correspond to rows 4 and 5 in Figure 2B). Putting these two parts together, now for every position *t* in the input alignment, we have the following joint probability (i.e., the complete likelihood) of the observed alignment block **A** *_t_*, the vector of instantiated extant functional states **z** *_t_*, and an instantiated ancestral functional state 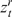 under a conditional shadowing model with multiple evolutionary trees (aka, CSMET):
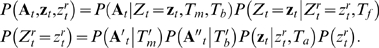
(2)In practice, the leaf functional states **z** *_t_* of an alignment block starting at position *t*, and the ancestral functional state 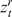 are not observed. Thus the likelihood score of **A** *_t_* follows a complex mixture of *marginal* phylogenies defined by all possible joint configurations of functional states 
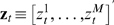
 and the ancestral state *z^r^*, rather than a simple motif/background mixture as in extant models. The typical tasks in motif detection involves either computing the marginal conditional likelihood 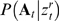 for all possible states of 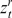, which will be used as the emission probability in an HMM of the ancestral functional states over the entire alignment (to be detailed in the next section); or the marginal posterior *P*(**z** *_t_*|**A**_1:*T*_), which will be used to extract the maximum *a posteriori* (MAP) motif annotation of the alignment. Both tasks involve a marginalization step that sums over all joint configurations of the internal tree nodes, *z_r_*'s, and **z** *_t_*'s. This leads to an inference problem in a state space defined by the product of multiple trees and therefore can be computationally intensive. Since in practice it is unusual to encounter more than 20 or so taxa in the comparative genomic setting, inference is still feasible. In this case, one can apply a *coupled-pruning algorithm* described in the Materials and Methods or a standard junction tree algorithm [31] for exact inference.

For an alignment of a large number of species and/or for a problem which involves searching for a large number of motifs simultaneously, marginalization of the product space of trees can be prohibitive. In these circumstances, we can apply an approximate inference method such as the generalized mean field algorithm [32], which decomposes the coupled trees in CSMET into disjoint trees and applies iterative message-passing across these trees to obtain an approximate posterior of **z** *_t_* or the conditional likelihood of **A** *_t_*. Alternatively we can replace some or all of the full phylogenetic trees for motif, background and functional evolution by star-topology phylogenies as in PhyloGibbs [9]. For simplicity of the exposition, we omit details of these generalizations.

#### Tree- and rate-transition along alignment

Different sites in the genome are subject to different evolutionary constraints and therefore follow phylogenetic trees with different equilibriums, topologies and rates. The conditional phylogenetic shadowing model described above couples multiple site-specific trees of all sites *within* a moving window of alignment block via a functional phylogeny; but it does not explicitly model transitions between possibly different evolutionary processes as the window scans over different functional entities along the aligned sequences, for example, transitions between motifs and different background regions, and among different motifs.

We introduce a hidden Markov model to model the transitions between functional annotations along the alignment. In principle, this HMM can employ highly structured transition models such as the global HMMs used in LOGOS [33] or CISTER [34], which intend to capture sophisticated “motif grammars” underlying higher eukaryotic CRMs. In this paper, we adopt a simplistic 3-state HMM that models the length of the spacer between motifs as a geometric distribution, and allows the motifs to be on either strand of the DNA. We define the HMM over the sequence of ancestral functional states 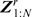, modeling the spatial transitions of functionalities along a hypothetical ancestral regulatory sequence underlying the aligned sequences from the study species. To model TFBS on either DNA strand with opposite orientations, two functional states are needed for each type of motifs, which determine the appropriate orientation for the PWM employed by the motif tree *T_m_* for defining the likelihood of a selected DNA substring; but these two functional states correspond to a degenerated motif state (i.e., 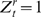) at the root of the functional tree *T_f_* in CSMET, and follow the same turnover process. Details of such an HMM is given in Materials and Methods.

Unlike the standard HMM for mono-genomic motif detection where the emission probability uses a simple conditional multinomial distribution of a single nucleotide, or a PhyloHMM for comparative-genomic motif detection ignoring motif turnover where the emission probability is defined by a conditional likelihood of a column of aligned nucleotides under a single phylogeny, to accommodate functional turnover of segments in certain species in the alignment, we define the emission model to be the CSMET conditional likelihood of an alignment block, 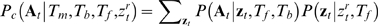, and thereby enable conditional shadowing over the taxa at each site. A technical issue arising from this construction is that unlike the PhyloHMM, which is still a standard 1st-order HMM, in our case we have a higher-order HMM due to the contex-dependent coupling of all the sites within a motif by the functional phylogeny *T_f_*, which models the whole sequence segment within a window of length *L* as a unit. In the next section, we outline statistical inference strategies that address this technical issue.

### Strategy

#### Posterior inference

**Figure 3:**
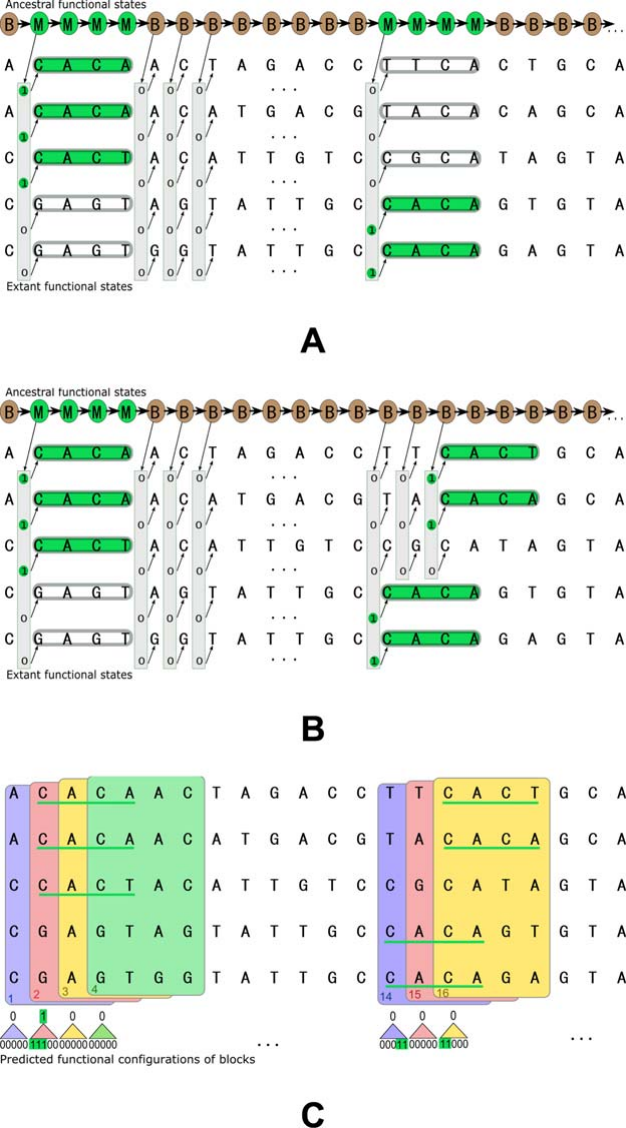
Context-specific relationships among variables due to two possible value configurations of hidden variables shown in (A) and (B). Note that when the ancestral state is a motif, the segment corresponding to the TBFS evolves as a unit (as shown by the arrow from an extant functional state pointing to a multi-column segment), either retaining its functionality as a motif, or turning-over to a background segment, as illustrated in (A). When the ancestral state is a background, then every position can evolve independently as long as it is still in the background (as shown by the arrow from a functional state pointing to a single column). But when a motif emerges out of the background, as shown in (B), the segments corresponding to the TFBS start to evolve as a unit, causing even the aligned nucleotide positions to evolve under different positional constraints. (C) Outlines the idea of a block approximation of the CSMET emission probability.

The incorporation of the functional phylogeny *T_f_* to explicitly model functional turnover of entire segments (rather than individual sites) of DNA sequences in different taxa in a multiple alignment introduces not only higher-order Markov dependencies among sites, but also context-dependent dependencies among taxa. Thus CSMET is essentially a probabilistic model with *context-specific independencies*, which is well-known to be intractable in general [35]. Figure 3A and 3B show an example of the context-specific relationships among variables due to two different possible value-configurations of the hidden variables corresponding to ancestral and taxa-specific functional annotations (of a small chunk of the alignment). Computing the likelihood of the entire alignment requires a summation of all joint configurations of all of these hidden variables, for which no efficient exact algorithm resembling the dynamic programming algorithms applied to HMMs is available.

While it is possible to implement a Monte Carlo algorithm that performs sampling over the functional annotation space of 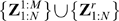 conditioning on the observed multiple alignment, we propose an approximate algorithm for posterior inference. As illustrated in Figure 3C, we can treat an *N*-column alignment as a sequence of (*N*−*L*+1) consecutive *L*-column aligned blocks. We assume each such block **A** *_t_* is either generated from a CSMET emission model conditioning on the ancestral function of this segment being a background, i.e., 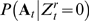, or it can be generated from a CSMET conditioning on the ancestral function being a motif, say, of type *k*, expressed as 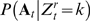. We can pre-compute the emission probabilities for all the aligned blocks, plug them back into an equivalent HMM of 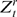
's on blocks rather than on columns, and then compute the posterior probabilities or Viterbi-sequence of the labels of each block using the standard dynamic programming algorithms (e.g., forward-backward) for HMMs (see Materials and Methods section for details). The approximation introduced here lies in the approximate computing of the emission probabilities for the blocks, specifically at the boundary between motifs and background. For these blocks the likelihood of the aligned sequences should be defined by two different emissions, one on the background sub-block and the other on the motif sub-block, whereas our approximation employs only a single emission—either an entirely background-derived CSMET 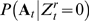 or an entirely motif-derived CSMET 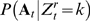. But since our approximation results in a poorer fit only for the boundary regions, we expect that the overall posterior indication of the motifs, which is primarily driven by the emission probabilities of the motif blocks, will only suffer moderate weakening of contrast at the boundaries. We refer to this approximation method as *block-approximation* (BA). Another more subtle approximation due to BA is the ignoring of different turnover behaviors within a block **A** *_t_* conditioning on the ancestral function of this segment( being a motif or a background), as exemplified in Figure 3B. Unlike a motif block derived from an ancestral motif, a segment of ancestral background sites do not evolve as a whole block, thus a block **A** *_t_* entirely originated from a ancestral background can contain rows (descendents) that are either entirely non-motif, or partially non-motif and partially motif (i.e., starting from an arbitrary position *t′* in window *t*:*t*+*L*, the segment *t′*: *t′*+*L*, part of which extends out of **A** *_t_*, in an arbitrary taxon can evolve into a motif), whereas a block **A** *_t_* entirely originated from a motif can only contain either fully preserved motif rows or turned-over non-motif rows. A detailed discussion of this subtlety is beyond the scope of this paper, and BA simply treats each entire row in **A** *_t_* as a homogeneous functional evolutionary unit. The computational time for BA is linear in the length of the input, with a multiplicative factor determined by the length of the motif and the number of species concerned in the alignment. In case of multiple motifs, the emission probabilities of the blocks should be computed under the unique CSMET of each motif. Since motifs can have different lengths, bookkeeping of all the emissions can be slightly more complicated due to the need to handle blocks of different lengths. But the computational cost is only increased by the order of the number of the motifs in question. For simplicity, we defer details of this generalization to a later update of the CSMET.

With the BA strategy, we arrive at an approximation to the posterior distribution of motif annotation at every position given the entire alignment, 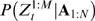, and the posterior of the sequence of ancestral functions, 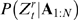. For an alignment block of which only a few taxa correspond to motifs and others are merely background, under the CSMET model, the 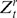 of this block can be either motif or background. In the first case, it means that absence of motifs in some taxa is interpreted as the result of loss of ancestral motifs, whereas in the second case, the presence of motifs in some taxa is interpreted as the result of emergence of nascent motifs out of the background. As far as we are aware of, CSMET is the only motif-finding algorithm that rigorously offers a closed-form deterministic solution to the posterior probability distribution of motif annotations both in the alignment and in the ancestral sequence over the entire space of binding site configurations. PhyloGibbs [9] offers a sample-based solution to the posterior of 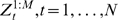 given **A**_1:*N*_, but as mentioned earlier, it is not based on an explicit model of binding site turnover, and thus does not have a closed-form expression that can motivate efficient deterministic approximation.

#### Maximum likelihood training

The CSMET can be trained on annotated CRM alignments. We need to learn the nucleotide phylogenetic trees for motifs and backgrounds, and the phylogenetic tree that describes the evolution of functional annotation. We use the F84 model for nucleotide substitution on the motif and background trees; for evolution of functional annotation, we use the simpler JC69 model. As detailed in Materials and Methods and Text S1, for a given tree topology, for the JC69 model all we need to estimate is the branch length on the tree, which relates to total substitution probability. For the F84 model, besides the tree topology, we need to estimate the stationary distribution, which we set to be the PWM for motif phylogenies or the background nucleotide frequencies for background phylogeny; and also two additional evolutionary parameters: the overall substitution rate per site *μ* and the transition/transversion ratio *ρ*.

Given a multiple alignment, the ground truth of functional annotation, the PWMs for motifs, and nucleotide frequency for the background, we use the following strategy for estimating the trees and the evolutionary parameters. Detailed derivation and explicit expressions are provided in Materials and Methods.

- Find a tree topology *τ* and the branch lengths *β* by running fastDNAml [36] over the entire alignment.
- Find a scaling factor *r_f_* over branch lengths *β_f_* of the functional tree *T_f_*, by maximizing the likelihood of aligned functional annotations under *T_f_* via a line-search in parameter space.
- Find a scaling factor *r_m_* over branch lengths *β_m_* of the motif tree *T_m_*, and the Felsenstein rate *μ_m_*, by maximizing the likelihood of aligned motif sequence under *Tm* with the F84 model.
- Find a tree topology *τ_b_* and branch lengths *b*_0_ for background tree *T_b_* by running fastDNAml directly over only the background sequences. The Felsenstein rate *μ_b_* is then estimated by maximizing the likelihood under *T_b_* with a simple line-search.

To compute the Felsenstein substitution rate *μ*, we use a fixed transition-transversion ratio of 2. If the stationary nucleotide distribution defined by the motif PWM is incompatible with this value of the transition-transversion ratio, we set it to the smallest value that is compatible with the stationary distribution as in [5].

### Performance on Synthetic Data

At present, biologically validated orthologous motifs and CRMs across multiple taxa are extremely rare in the literature. In most cases, motifs and CRMs are only known in some well-studied *reference taxa* such as the *Drosophila melanogaster*; and their orthologs in other species are deduced from multiple alignments of the corresponding regulatory sequences from these species according to the positions and PWMs of the “reference motifs” in the reference taxon. This is a process that demands substantial manual curation and biological expertise; rarely are the outcomes from such analysis validated *in vivo* (but see [8] for a few such validations in some selected *Drosophila* species where the transgenic platforms have been successfully developed). At best, these real annotations would give us a limited number of true positives across taxa, but they are not suitable for a systematic performance evaluation based on precision and recall over true motif instances. Thus we first compare CSMET with a carefully chosen collection of competing methods on simulated CRM sequences, where the motif profiles across all taxa are completely known.

We choose to compare CSMET with 3 representative algorithms for comparative genomic motif search, PhyloGibbs, EMnEM, PhyloHMM; and the program Stubb, which is specialized for motif search in eukaryotic CRMs, and in our paper, set to operate in mono-genomic mode. The rationale for choosing these 4 benchmarks is detailed in the Material and Methods.

#### Multi-specific CRM simulator

**Figure 4:**
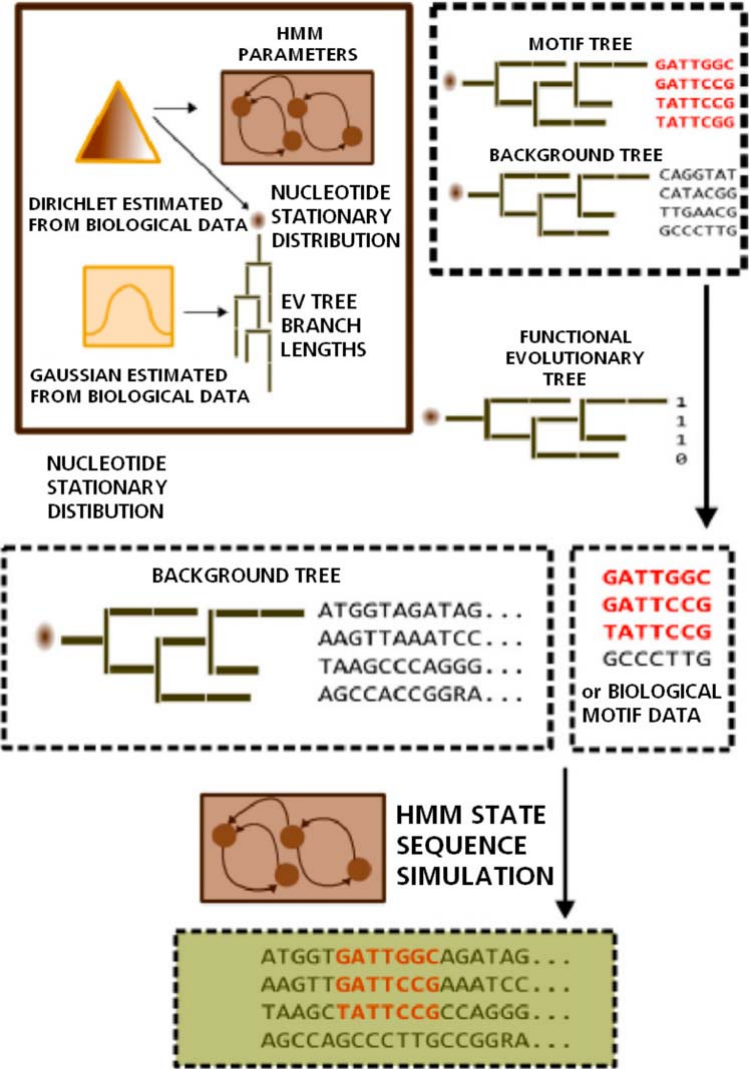
An illustration of the generative scheme of a Multi-specific CRM simulator.

We developed a simulator of multi-specific CRMs with flexible TFBS turnover dynamics across taxa and realistic TFBS arrangement within CRM. The overall scheme is illustrated in Figure 4. Specifically, the input of the simulator includes: 1) topologies of the phylogenetic trees for nucleotide (e.g., in motif sites and background) and functionality substitutions; 2) prior distributions of the stationary distribution of states (i.e., nucleotide or functionalities) at the roots of the trees; 3) prior distributions of the branch lengths of the trees and the substitution rates, and other evolutionary parameters where necessary (e.g., the Felsenstein rate *μ* and *ρ* in F84 model); 4) a global HMM encoding the motif grammar in the CRMs. As detailed in the Material and Methods, during simulation, all building blocks of a CRM, such as the motif instances, background sequences, functionality states (that determines motif turnover) in different taxa, and positions of the motifs in the CRM are sampled separately as illustrated in Figure 4, and put together to synthesize an artificial CRM. This simulator can be used to simulate realistic multi-specific CRMs resulting from various nontrivial evolutionary dynamics. It is useful in its own right for consistence/robustness analysis of motif evolution models and performance evaluation of comparative genomic motif-finding programs.

Below, we report results of four experiments based on simulated datasets. Each experiment was based upon varying one parameter of the model, keeping all the others fixed, in order to analyze robustness of CSMET and various other methods under different conditions. Every simulated CRM alignment contained 10 taxa, and for each experiment we simulated 50 datasets. The simulated data is available at the CSMET website to allow external comparisons. Performance of all the tested programs were based on the precision, recall and their F1 score (i.e., the harmonic mean of precision and recall) [37].

#### Performance under varying degrees of motif turnover

To examine the effect of motif turnover (i.e., functional conservation) in aligned regions across taxa on the motif-detection performance, we simulated CRM alignments with differing magnitudes of the evolutionary rate along the functional phylogeny. Since known motifs in the Drosophila species we are working with usually have around 75% conservation, we chose our evolutionary rates so as to achieve conservation percentages between 64%–75% (or equivalently, turnover percentages between 25%–36%) at the species-specific motif-instance level. (See Text S1 for how this is achieved.)

**Figure 5:**
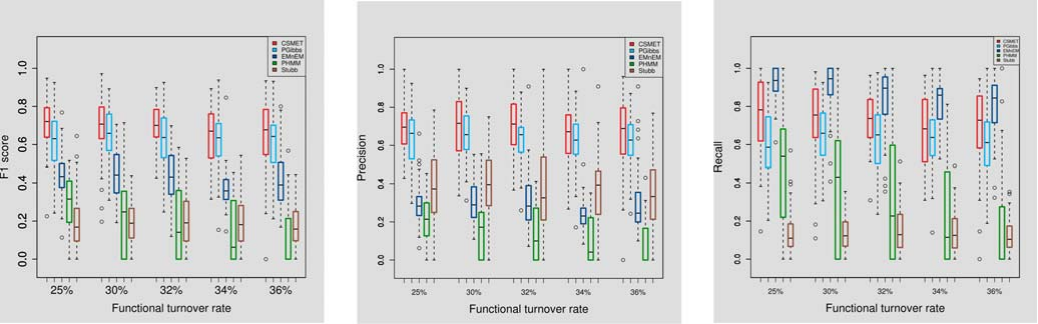
Performance under varying degrees of functional conservation.

We find that even with increasing rates of functional turnover, the performance of CSMET and Phylogibbs remain largely stable, with CSMET consistently dominating PhyloGibbs in F1 score with a modest margin (Figure 5). The margin is statistically significant with *p* = 2.48×10^−7^ under a paired *t*-test. EMnEM has a high recall score, but overall its F1-scores are well below CSMET and PhyloGibbs, also it appears to be affected more by the increased turnover rates. PhyloHMM shows an interesting trend, it performs better than its non-phylogenetic cousin Stubb on data with low turnover rates, but its performance worsens when compared to Stubb on data with increasing turnover rate. This shows that a naive application of phylogenetic shadowing in multi-species alignment with high functional divergence can actually result in degraded performance compared even to just single species analysis.

#### Performance under varying degrees of motif/background contrast

**Figure 6:**
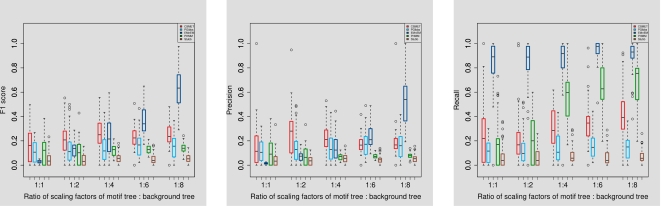
Performance under varying degrees of motif/background contrast.

The difference in conservation between the motif and background sequences will have an impact on the performance of the model. However, this experiment can be performed in two different ways: changing the degree of similarity between motif and background stationary distributions; and changing the evolutionary rates of one or the other. We choose the second method and conduct the simulation as follows: we attribute the motif phylogeny with a low entropy stationary distribution resembling a PWM, and with a fixed evolutionary rate; and we let the background to have a stationary distribution similar to but with higher entropy than that of the motif, and have a variable evolutionary rate. The evolutionary rate in the background tree is changed gradually from low values to high values, by varying the scaling factor applied to the background tree from 1 to 8. This is to check how well the CSMET model may detect motifs emerging out of the background with differing degrees of sequence-level conservation with respect to the background caused by their relative evolutionary rates. The corresponding performances are shown in Figure 6.

We found that even under low variation between the motif and background, i.e., both following an evolutionary tree with similar stationary distribution, and the same branch lengths and scaling parameters, CSMET outperforms all the other methods. CSMET steadily improves in performance upto the scaling factor of 4, after which its performance roughly plateaus. PhyloGibbs behaviors similarly, but overall with lower F1 scores that is statistically significant (*p* = 1.41×10^−14^). EMnEM, on the other hand outperforms all other methods for scaling factors of 6 or more; meaning that when motifs are extremely highly conserved compared to the background, the advantage of modeling their turnover as in CSMET and PhyloGibbs over using a basic phylogenetic model diminishes, which is well expected. Since in real CRMs, the evolutionary rates of of the non-functional regions with respect to that of the functional regions (e.g., coding regions, TFBSs) in eukaryotes have been shown to be lie between 1.2 and 2.5 [1], we can claim that CSMET outperforms all other software in the region of biologically relevant parameter settings.

### Robustness on Data Violating CSMET Model Assumptions

#### Effect of non-uniform functional evolution rates

**Figure 7:**
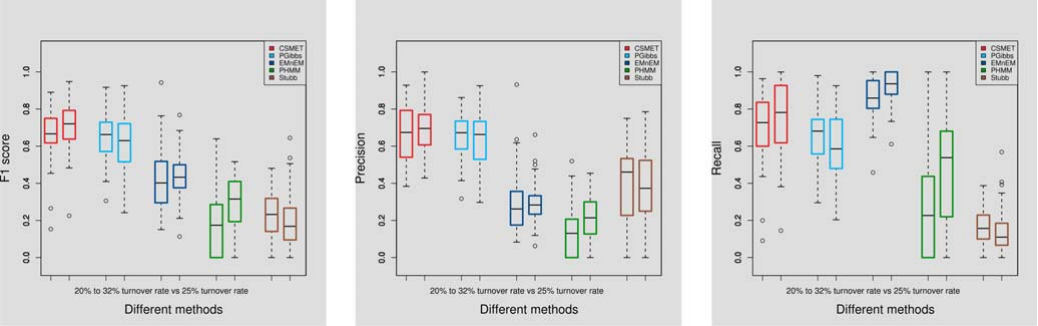
Effect of varying motif turnover rates across sequence. In the pair of barplots of each method, the left bar corresponds to performance with varying turnover rates ranging from 20% to 32%; the right bar corresponds to performance under a fixed turnover rate at 25%.

We analyzed the robustness of CSMET (compared to other algorithms) in the face of a breakdown of a key CSMET model assumption—that the motif turnover rates are allowed to vary along the simulated CRM sequences instead of staying constant, which is possible in real regulatory sequences. The CSMET model does not explicitly address this dynamics and simply assumes an invariant turnover rate throughout the sequence. We simulated a dataset where the motif turnover rates are chosen uniformly from 4 pre-specified categories, corresponding to branch scaling factors of 1.00, 1.50, 2,00 and 2.50, respectively, over the baseline phylogeny. The corresponding motif turnover rates were 20%, 25%, 30% and 32%, respectively. As shown in Figure 7, we found that while performance of CSMET on such data declines compared to its performance on data simulated with a invariant turnover rate, it still performs no worse than any of the other software even though a primary assumption it adopts (that of a constant functional turnover rate) is violated.

#### Effect of different generative model

To examine the robustness of CSMET under the violation of many of its model assumptions all at the same time, we then performed an experiment using an external simulator PSPE [22], which is based on an entirely different generative model with respect to CSMET (in terms of nucleotide substitution, motif placement, motif turnover, etc.) to synthesize multi-specific CRM sequences. However, at times PSPE generates motifs in some species with some lateral displacements, which appears to be an empirical operation not universal to evolutionary mechanisms that lead to functional turnover in *aligned* motifs (e.g., see [12]), but similar to an assumption underlying PhyloGibbs. To obtain a fair comparison, we suppress the lateral displacements by a post-processing of the sequences simulated by PSPE. In the post-processing step, we remove any motif instances that are laterally displaced in the multiple sequence alignment that is generated. This leaves us with a multiple sequence alignment with all the motif instances perfectly aligned.

**Figure 8:**
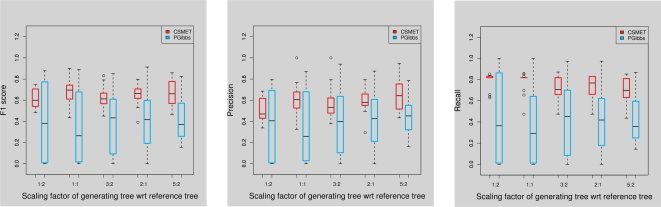
Performance on modified PSPE data. The label on the *x*-axis denotes the scaling factor used by the PSPE tree with respect to a reference *Drosophila* phylogeny.

We used PSPE driven by five different scaled versions of the phylogenetic tree on the 11 *Drosophila* species to simulate different degrees of motif evolution, and test CSMET and PhyloGibbs on simulations under each scaled tree. For sequence evolution, an HKY nucleotide substitution model with parameter set to 0.05 was used; for the gap distribution, a negative binomial distribution with parameters {1, 0.5} was used (note that none of these assumptions are used in CSMET). The motif sequence was generated by PSPE from the default constraints provided. We generated sequences of length 1000 for training, each with about 7–10 motifs; and we test on sequences of length 500 containing 4–5 motifs. For each tested simulation condition (i.e., tree scaling factor), 50 samples were generated, and the performance of CSMET and PhyloGibbs are shown in Figure 8. We can see that the F1 scores of CSMET are quite stable under different tested conditions and with low variance, and in all conditions CSMET outperforms Phylogibbs on F1 scores, and the margins are statistically significant (*p* = 1.875×10^−13^). This suggests that CSMET is reasonably robust with respect to violations of its model assumptions.

### Performance on Aligned *Drosophila* CRMs

We applied CSMET and competing methods to a multi-specific dataset of *Drosophila* early developmental CRMs and motifs compiled from the literature [38]. However, in this situation, we score accuracy only on the motifs annotated in *Drosophila melanogaster* (rather than in all taxa), because they are the only available gold-standard. Upon concluding this section, we also report some interesting findings by CSMET of putative motifs, some of which only exist in other taxa and do not have known counterparts in *melanogaster*.

#### Real CRMs from 11 *Drosophila* taxa

To evaluate CSMET on real sequence data, we use a pre-aligned benchmark data set containing multiple alignments of orthologous CRMs from 11 related *Drosophila* species, whose divergence time with respect to the most recent common ancestor is roughly 50 million years. The species included are: *melanogaster*, *simulans*, *sechellia*, *yakuba*, *erecta*, *ananassae*, *persimilis*, *pseudoobscura*, *mojavensis*, *virilis*, and *grimshawi*. Our data set contains 14 different multiple-alignments ranging from 3640-bp to 5316 bp long; each alignment corresponds to a DNA segment containing a CRM (Table 1) that has been annotated in *Drosophila melanogaster* [38],[39] plus 1000bp flanking regions on both ends, and its putative orthologs in the other 10 taxa identified using the precompiled *Drosophila* genome data from the UCSC Genome browser website [40]. Overall, our data set contains 250 instances of motifs in a total of 14 CRMs. To our knowledge, it represents one of the most complete multi-genomic collection of *Drosophila* CRM/motifs. This dataset, along with a full graphical representation of the CRMs and TFBSs, are available at the CSMET website.

**Table 1:** A short summary of the nature of the annotated CRMs.

Name of CRM	Length	Motif types
Abdominal A	1745	Hunchback, Kruppel
Buttonhead	1429	Bicoid, Hunchback
Engrailed	900	Caudal
Eve Str 2	730	Bicoid, Hunchback, Kruppel
Eve Str 3+7	512	Hunchback, Knirps
Eve Str 4+6	602	Hunchback, Knirps
FushiTarazu Zebra	653	Caudal
Hairy Str 5	1574	Kruppel
Hairy Str 6	556	Caudal, Hunchback, Knirps, Kruppel
Hairy Str 7	1471	Bicoid, Hunchback, Kruppel
Kruppel 730	1158	Bicoid, Hunchback, Knirps
Runt	1335	Bicoid, Hunchback, Knirps, Kruppel
Spalt	721	Bicoid, Caudal, Hunchback, Kruppel
Tailless	635	Caudal, Bicoid

#### Results on real CRM data sets

**Figure 9:**
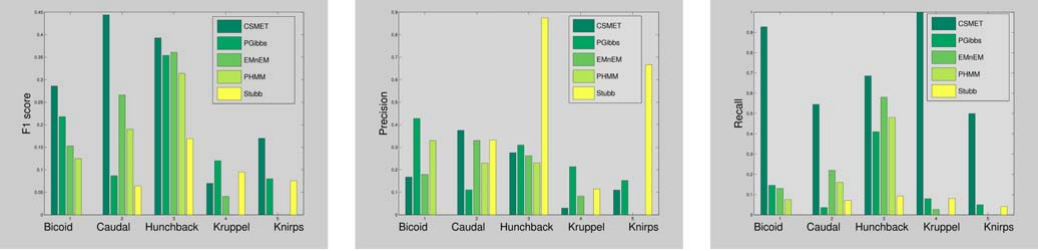
Comparison of algorithms on motif search performance over 5 motifs on real CRMs.

Using a 1 versus *K*−1 cross validation scheme detailed in the Materials and Methods section, where *K* is the total number of CRMs in which a motif in question is present, we tested all algorithms on five motifs, *Bicoid*, *Caudal*, *Hunchback*, *Kruppel* and *Knirps*, one motif type at a time, and the results are summarized in Figure 9. We used posterior decoding for CSMET and PhyloHMM, since even motifs of the same type can overlap on opposite strands or even on the same strand. For the other three algorithms, we explored their optimum parameter configuration to get meanful results. The five algorithms were compared on the basis of precision, recall, and their F1 score only on the *melanogaster* motifs they manage to identify within the CRMs. Overall, CSMET outperforms all other methods in all motifs except for Kruppel. For Kruppel, all methods perform poorly because the quality of the PWM that can be obtained from training data has very high entropy. Figure 9B and 9C also show that CSMET gives a much higher recall score than other softwares in most cases while maintaining a precision comparable to them (except in some cases where Stubb has very high precision but very low recall). It is worth mentioning that in these real CRMs, biological annotations tend to be conservative because they are only based on existing footprinting experiments performed in a non-exhaustive fashion in most of the CRMs. Thus a high recall is not very surprising.

**Figure 10:**
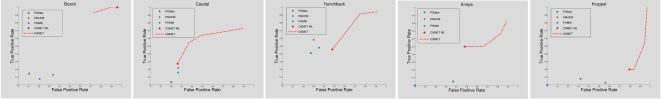
ROC of CSMET with different values of functional evolutionary (i.e., TFBS turnover) rates on *Drosophila* CRMs.

Since real CRM data are more complex than simulated data due to the presence of a significant number of gaps, broken motifs etc., there is a significant variance in the performances across different motifs by CSMET, as well as by all other algorithms; on the other hand, training data for fitting the model parameters needed in a CSMET is extremely limited. We found that the performance of CSMET can be improved over its maximum likelihood configuration (determined from training data) by adjusting the values of the evolutionary parameters. The evolutionary parameters that are estimated from the training data are: the tree evolutionary rates (represented as the scaling coefficients of the tree branches) for the motif and annotation tree, and the Felsenstein rates for the motif and background nucleotide substitution models. Of these parameters, we found that the predictive power of the model is most significantly affected by the evolutionary rate of the functional tree. Figure 10 shows the ROC curve of CSMET performance under various values of the evolutionary rate *r* ranging from a half to 4 times the maximum likelihood estimator of *r*, along with the scores of 3 competing softwares at a working parameterization adjusted based on their default setting. From Figure 10, it is noteworthy that the performance of all programs on the Hunchback motif is generally good. This is probably because the *Hunchback* motif instances are generally very well conserved, and thus the quality of our training annotation based upon visual inspection is relatively more reliable.

#### Findings on real CRM data sets

**Figure 11:**
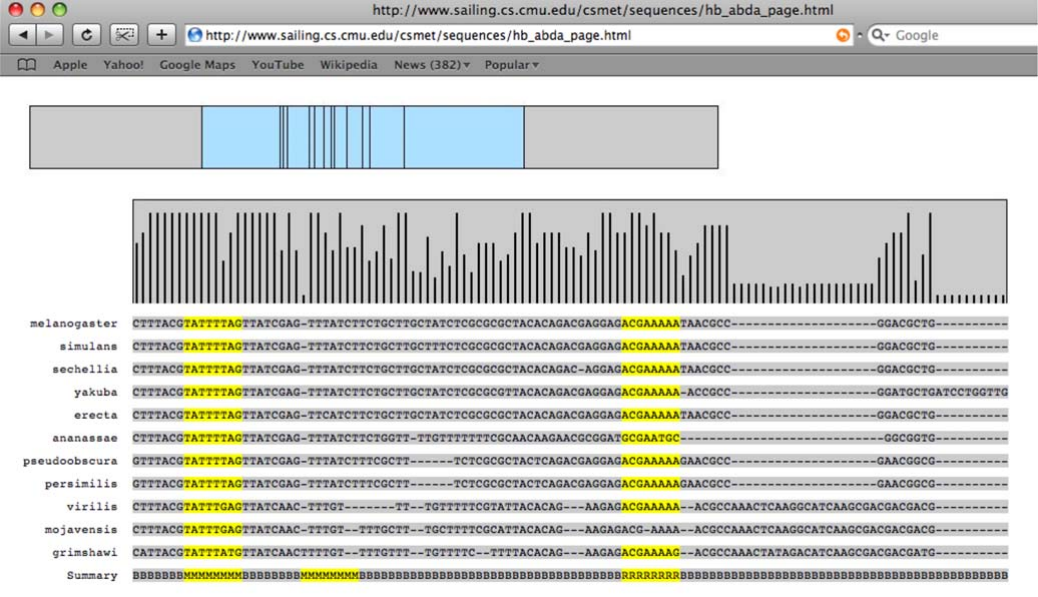
A screenshot of the summary of TFBS-predictions as displayed on the CSMET website.

**Figure 12:**
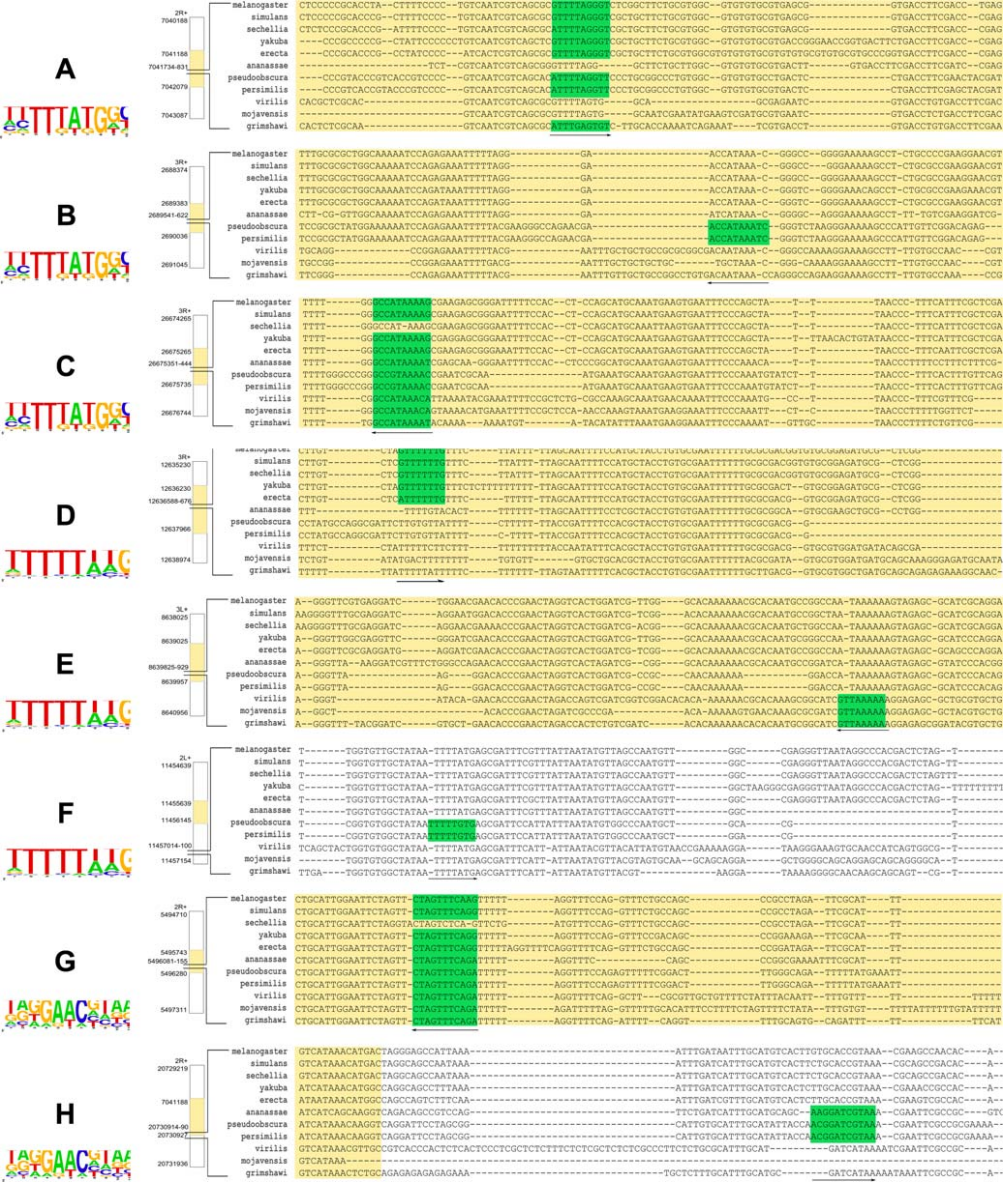
Example of previously unknown or biologically validated motif instances uncovered by CSMET in the presence of functional turnover or misalignment. CRM regions are shown in yellow in the alignment. The genomic loci for the flanking region borders, CRM borders and display snippet borders for *melanogaster* assembly 4 are shown on the immediate left of the alignment; with the logos of the identified motifs shown on the far left [43]. (a) A *Caudal* motif in *Engrailed* CRM Alignment. (b) A *Caudal* in *FushiTarazu Zebra* CRM. (c) A *Caudal* in *Tailless*. (d) A *Hunchback* in the *Abda* CRM. (e) A *Hunchback* in *Hairy stripe7* CRM. (f) A *Hunchback* in *Spalt* CRM flanking region about 1000 bp apart from the CRM. (g) A *Knirps* in *Even skipped stripe 4/6* CRM. (h) A *Knirps* in *Kruppel 730* CRM flanking region 38bp apart from the CRM.

CSMET has correctly retrieved a significant portion of previously known TFBSs within the 14 CRMs in the *melanogaster* taxon, along with their putative conserved orthologs in other taxa, or in some cases, apparent site turnovers in other taxa. Furthermore it has also found numerous interesting instances of alignment blocks of putative TFBSs not known before, both inside CRMs as well as in CRM flanking regions, where TFBS turnovers are apparent in some taxa. A database containing the complete summary of our predictions is available at http://www.sailing.cs.cmu.edu/csmet/, where the positions and taxonomic-identities of all predicted TFBSs and turnovers are documented graphically with appropriate color highlights for each of the 14 CRM alignments we analyzed. Figure 11 shows a snapshot of a fraction of one of the annotated alignments in our database. Some examples of the predicted TFBSs are presented in Figure 12.

Due to the functional heterogeneity across taxa in many of these alignment blocks of putative TFBSs, these motifs can be difficult for other algorithms to detect. Some of these instances correspond to putative TFBSs appearing in non-melanogaster taxa, such as the putative Knirps motif block in the Kruppel 730 CRM (Figure 12H), and the putative Hunchback motif in the flanking region of Spalt CRM (Figure 12F). Another interesting observation is that numerous putative TFBS blocks were identified not just inside the developmental CRMs but also in the flanking regions of the CRMs we analyzed. We had chosen 1000 bp of flanking region from *D. melanogaster*, and found that while some putative sites are located within 100 bp of established CRM boundaries (e.g., Figure 12H), others may lie as far away as 1000 bp (our limit of analysis) and possibly further away from established CRM boundaries (e.g., Figure 12F). We also noted several interesting patterns in examples of functional turnover. These include single species loss of TFBSs, as for the *Caudal* motif in the Tailless CRM region (Figure 12C) and for the *Knirps* motif in Even Skipped Stripes 4+6 CRM region (Figure 12G); and subclade specific loss or gain of binding sites, as in the *Hunchback* motif block in the Abdominal A CRM region (Figure 12D) and the *Hunchback* motif block in the Hairy Stripe 7 CRM region (Figure 12E). A common form of subclade specific loss or gain is that they take place in closely related sister taxa, like *D. pseudoobscura* and *D. persimilis* as in the *Caudal* motif in the Fushi Tarazu Zebra CRM (Figure 12B) and the *Hunchback* motif in the Spalt CRM (Figure 12F).

To assess whether CSMET predicts TFBSs of biological significance, we tried validating our findings by checking which of our predicted motif blocks with functional turnover had been biologically validated. While this is not possible for motifs predicted only in non-melanogaster taxa, or for motifs predicted in CRM flanking regions, we found numerous examples of conserved motif blocks which were biologically validated for the ortholog in *D. melanogaster*. For example, based on the binding site database of papatsenko, the *Caudal* motif block in Tailless CRM (Figure 12C) and the *Hunchback* block in Abdominal A CRM (Figure 12D) were both biologically validated. We further used two recently available large public TF databases—Oreganno [41] and the RegFly [42]—to check if we could find biologically validated binding sites outside those listed in papatsenko. Of the 8 motifs displayed, 2 additional cases were confirmed in this independent dataset—the *Caudal* motif in the Fushi Tarazu Zebra enhancer (Figure 12B) region, and the *Hunchback* in the Hairy Stripe 7 (Figure 12E) region. Even though we did not perform an exhaustive search to examine whether the validated binding sites (with functional turnover in other species) predicted by CSMET were also predicted by other programs, our results include several non-conserved biologically validated binding sites which are predicted by CSMET but not by PhyloGibbs, including the *Hunchback* motif in Abdominal A CRM (Figure 12D), and the *Hunchback* motif in Hairy Stripe 7 CRM (Figure 12E). Other such binding sites like mel3L+:8639083 were also noted.

## Discussion

CSMET is a novel phylogenetic shadowing method that can model biological sequence evolution at both nucleotide level at each individual site, and functional level of a whole TFBS. It offers a principled way of addressing the problem that can seriously compromise the performance of many extant conservation-based motif finding algorithms: motif turnover in aligned CRM sequences from different species, an evolutionary event that results in functional heterogeneity across aligned sequence entities and shatters the basis of conventional alignment scoring methods based on a single function-specific phylogeny. CSMET defines a new evolution-based score that explicitly models functional substitution along the phylogeny that causes motif turnover, and nucleotide divergence of aligned sites in each taxa under possibly different function-specific phylogenies conditioning on the turnover status of the site in each taxon.

In principle, CSMET can be used to estimate the rate of turnover of different motifs, which can elucidate the history and dynamics of functional diversification of regulatory binding sites. But we notice that experimentally validated multi-species CRM/TFBS annotations that support an unbiased estimate of turnover rates are yet to be generated, as currently almost all biologically validated motifs only exist in a small number of representative species in each clade of the tree of life, such as *melanogaster* in the *Drosophila* clade. Manual annotation on CRM alignments, as we used in this paper, tends to bias the model toward conserved motifs. Thus, at this time, the biological interpretation of evolutionary parameters on the functional phylogeny remains preliminary. Nevertheless, these estimated parameters do offer important utility from a statistical and algorithmic point of view, by elegantly controlling the trade-off between two competing molecular substitution processes—that of the motif sequence and of the background sequence—at every aligned site across all taxa beyond what is offered in any existing motif evolution model. Empirically, we find that such modelling is useful in motif detection.

On both synthetic data and 14 CRMs from 11 *Drosophila* taxa, we find that the CSMET performs competitively against the state-of-the-art comparative genomic motif finding algorithm, PhyloGibbs, and significantly outperforms other methods such as EMnEM, PhyloHMM and Stubb. In particular, CSMET demonstrates superior performance in certain important scenarios, such as cases where aligned sequences display significant divergence and motif functionalities are apparently not conserved across taxa or over multiple adjacent sites. We also find that both CSMET and PhyloGibbs significantly outperform Stubb when the latter is naively applied to sequences of all taxa without exploiting their evolutionary relationships. Our results suggest that a careful exploration of various levels of biological sequence evolution can significantly improve the performance of comparative genomic motif detection.

Recently, some alignment-free methods [19] have emerged which search for conserved TFBS rich regions across species based on a common scoring function, e.g., distribution of word frequencies (which in some ways mirrors the PWM of a reference species). One may ask, given perhaps in the future a perfect search algorithm (in terms of only computational efficiency), do we still need explicit model-based methods such as CSMET? We believe that even if exhaustive search of arbitrary string patterns becomes possible, models such as CSMET still offer important advantage not only in terms of interpretability and evolutionary insight as discussed above, but possibly also in terms of performance because of the more plausible scoring schemes they use. This is because it is impractical to obtain the PWM of a motif in species other than a few reference taxa, thus the scores of putative motif instances in species where their own versions of the PWM are not available can be highly inaccurate under the PWM from the reference species due to evolution of the PWM itself in these study species with respect to the PWM in the reference species. The CSMET places the reference PWM only at the tree root as an equilibrium distribution; for the tree leaves where all study species are placed, the nucleotide substitution model along tree branches allows sequences in each species to be appropriately scored under a species-specific distribution that is different from the reference PWM, thereby increasing its sensitivity to species-specific instantiations of motifs.

A possible future direction for this work lies in developing better approximate inference techniques for posterior inference under the CSMET model, especially under the scenarios of studying sequences from a large clade with many taxa, and/or searching for multiple motifs simultaneously. It is noteworthy that our methods can be readily extended for *de novo* motif detection, for which an EM or a Monte Carlo algorithm can be applied for model-estimation based on the maximum likelihood principle. Currently we are exploring such extensions. Also we intend to develop a semi-supervised training algorithm that does not need manual annotation of motifs in other species on the training CRM alignment, so that we can obtain a less biased estimate of the evolutionary parameters of the CSMET model.

A problem with most of the extant motif finders, including the proposed CSMET, is that the length variation of aligned motifs (e.g., alignments with gaps) cannot be accommodated. In our model, while deletion events may be captured as gaps in the motif alignment, insertion events cannot be captured as the length of the motif is fixed. This is because in a typical HMM sequence model the state transitions between sites within motifs are designed to be deterministic. Thus stochastically accommodating gaps (insertion events) within motifs is not feasible. Hence, some of the actual motifs missed by the competing algorithms were “gapped” motifs. These issues deserve further investigation.

## Materials and Methods

### The Molecular and Functional Substitution Model

We use the Felsenstein 1984 model (F84) [29], which is similar to the Hasegawa–Kishino–Yano's 1985 model (HKY85) [44] and widely used in the phylogenetic inference and footprinting literature [5],[29], for nucleotide substitution in our motif and background phylogeny. Formally, F84 is a five-parameter model, based on a stationary distribution *π* ≡ [*π_A_*, *π_T_*, *π_G_*, *π_C_*]′ (which constitutes three free parameters as the equilibrium frequencies sum to ) and the additional parameters *κ* and *ι* which impose the transition/transversion bias. According to this model, the nucleotide-substitution probability from an internal node *c* to its descendent *c*′ along a tree branch of length *b* can be expressed as follows:
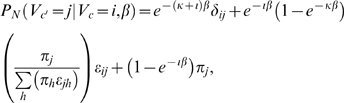
(3)where *i* and *j* denote nucleotides, *δ_ij_* represents the Kronecker delta function, and *ε_ij_* is a function similar to the Kronecker delta function which is 1 if *i* and *j* are both pyrimidines or both purines, but 0 otherwise. The summation in the denominator concisely computes purine frequency or pyrimidine frequency. A more intuitive parameterization for F84 involves the overall substitution rate per site *μ* and the transition/transversion ratio *ρ*, which can be easily estimated or specified. We can compute the transition matrix *P_N_* from *μ* and *ρ* using Equation 3 based on the following relationship between (*κ*,*ι*) and (*μ*,*ρ*):
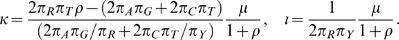
To model functional turnover of aligned substrings along functional phylogeny *T_f_*, we additionally define a substitution process over two characters (0 and 1) corresponding to presence or absence of functionality. Now we use the single parameter JC69 model [26] for functional turnover due to its simplicity and straightforward adaptability to an alphabet of size 2. The transition probability along a tree branch of length *β* (which represents the product of substitution rate *μ* and evolution time *t*, which are not identifiable independently,) is defined by:
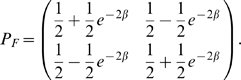
(4)We estimate the evolutionary parameters from training data based on maximum likelihood, details are available in the Text S1.

### Computing Complete- and Partial-Alignment Likelihood

A complete phylogenetic tree *T* ≡ {*τ*, *π*, *β*, *λ*} with internal nodes {*V_i_*; *i* = 1:*K′*} and leaf nodes {*V_i_*; *i* = *K′*+1:*K*}, where *K* denotes the total number of nodes (i.e., current and ancestral species) instantiated in the tree and the node indexing follows a breath-first traversal from the root, defines a joint probability distribution of all-node configurations (i.e., the nucleotide contents at an aligned site in all species instantiated in the tree), which can be written as the following product of nt-substitution probabilities along tree branches:
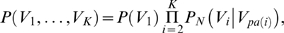
(5)where *V_pa_*_(*i*)_ denotes the parent-node of the node *i* in the tree, and the substitution probability *P_N_*() is defined by Equation 3. For each position *l* of the multiple alignment, computing the probability of the entire column denoted by *A_l_* of aligned nucleotides from species corresponding to the leaves of a phylogenetic tree *T* ^(*l*)^ defined on position *l*, i.e., *P*(*A_l_*|*T* ^(*l*)^), where *A_l_* correspond to an instantiation of the leaf nodes {*V_i_*; *i* = *K′*+1:*K*}, takes exponential time if performed naively, since it involves the marginalization of all the internal nodes in the tree, i.e.,
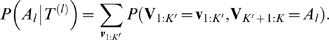
(6)We use the Felsenstein pruning algorithm [30], which is a dynamic programming method that computes the probability of a leaf-configuration under a tree from the bottom up. At each node of the tree, we store the probability of the subtree rooted at that node, for each possible nucleotide at that node. At the leaves, only the probability for the particular nucleotide instantiated in the corresponding taxon is non-zero, and for all the other nucleotides, it is zero. Unlike the naive algorithm, the pruning algorithm requires an amount of time that is proportional to the number of leaves in the tree.

We use a simple extension of this algorithm to compute the probabilities of a partial-alignment 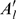 defined earlier under a marginal phylogeny, which is required in the coupled-pruning algorithm for CSMET, by considering only the leaves instantiated in 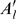 (but not in 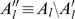) that is under a subtree *T′* ^(*l*)^ that forms the marginal phylogeny we are interested in. Specifically, let 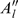 correspond to possible instantiations of the subset of nodes we need to marginalized out. Since we already how to compute *P*(*A_l_*|*T* ^(*l*)^) via marginalization over internal nodes 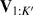, we simply further this marginalization over leaf nodes 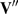 that corresponds to taxa instantiated in 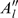, i.e.,
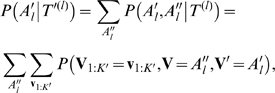
(7)where 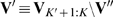 denotes the leaves instantiated in 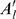. This amounts to replacing the leaf-instantiation step, which was originally operated on all leaves in the Felsenstein pruning algorithm, by a node-summation step over those leaves in 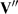. In fact, in can be easily shown that this is equivalent to performing the Felsenstein pruning only on the partial tree *T′* ^(*l*)^ that directly shadows 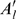, which is a smaller tree than the original *T* ^(*l*)^, and only requires time 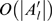.

### Computing the Block-Emission Probabilities

Under the CSMET model, to perform the forward-backward algorithm for either motif prediction or unsupervised model training, we need to compute the emission probability given each functional state at every alignment site. This is nontrivial because a CSMET is defined on an alignment block containing whole motifs across taxa rather than on a single alignment-column. We adopt a “block-approximation” scheme, where the emission probability of each state at a sequence position, say, *t*, is defined on an alignment block of length *L* started at *t*, i.e., 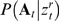, where **A** *t*≡(*A*_1_(*t*), *A*_2_(*t*),…, *A_L_*(*t*)), and *A_l_*(*t*) denotes the *l*th column in an alignment block started from position *t*.

The conditional likelihood **A** *_t_* given the nucleotide-evolutionary trees *T* and *T_b_* coupled by the annotation tree *T_a_* under a particular HMM state *s_t_* is also hard to calculate directly, because the leaves of the two nucleotide trees are connected by the leaves of the annotation tree (Figure 2B). However, if the leaf-states of the annotation tree are known, the probability components coming from the two trees become conditionally independent and factor out (see Equation 2). Recall that for a motif of length *L*, the motif tree actually contains *L* site-specific trees, i.e., 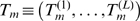, and the the choice of these trees for every site in the same row (i.e., taxon), say, 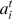 in the alignment block **A** *_t_*, is coupled by a common annotation state 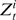. Hence, given an annotation vector *Z_t_* for all rows of **A** *_t_*, we actually calculate the probability of two subset of the rows given two subtrees (i.e., marginal phylogenies) of the original phylogenetic trees for motif and backgrounds, respectively (Figure 2B).

The subset 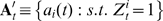 is constructed by simply stacking the DNA bases of those taxon for which the annotation variables indicate that they were generated from the motif tree.

The subtree 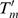 is constructed by simply retaining the set of nodes which correspond to the chosen subset, and the ancestors thereof. Similarly we have 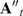 and 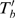. Hence, we obtain
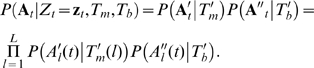
(8)


The probability of a particular leaf-configuration of a tree, be it a partial or complete nucleotide tree, or an annotation tree, can be computed efficiently using the pruning algorithm. Thus for each configuration of **z** *_t_*, we can readily compute 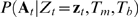 and 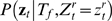. The block emission probability 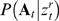 under CSMET can be expressed as:
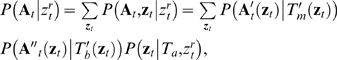
(9)


where we use 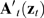, 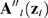, 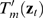 and 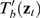 to make explicit the dependence of the partial blocks and marginal trees on functional indicator vector **z** *_t_*. We call this algorithm a *coupled-pruning algorithm*.

Note that in this algorithm we need to sum over a total number of 2*^M^* configurations of **z** *_t_* where *M* is the total number of taxa (i.e., rows) in matrix **A** *_t_*. It is possible to reduce the computational complexity using a full junction tree algorithm on CSMET, which will turn the graphical model underlying CSMET into a clique tree of width (i.e., maximum clique size) possibly smaller than *M*. But this algorithm is complicated and breaks the modularity of the tree-likelihood calculation by the coupled-pruning algorithm. In typical comparative genomic analysis, we expect that *M* will not be prohibitively large, so our algorithm may still be a convenient and easy-to-implement alternative to the junction-tree algorithm. Also this computation can be done off-line and in parallel.

### Posterior Inference Under CSMET

Given the emission probabilities for each ancestral functional state at each site, we use the forward-backward algorithm for posterior decoding of the sequence of ancestral functional states 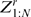 along the input CRM alignment of length *N*. The procedure is the same as in a standard HMM applied to a single sequence, except that now the emission probability at each site, say with index *t*, is defined by the CSMET probability over an alignment block **A** *_t_* at that position under an ancestral functional state 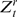, rather than the conditional probability of a single nucleotide observed at position *t* as in the standard HMM. The complexity of this FB-algorithm is *O*(*Nk* ^2^) where *k* denotes the total number of functional states. In this paper, we only implemented a simple HMM with one type motif allowed on either strand, so that *k* = 3. We defer a more elaborate implementation that allows multiple motifs and encodes sophisticated CRM architecture as in LOGOS [33] to a future extension.

Given an estimate of 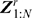, we can infer the MAP estimates of 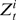
—the functional annotation of every site *t* in every taxon *i* of the alignment. Specifically, the posterior probability of a column of functional states *Z_t_* under ancestral functional state 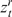 can be expressed as:
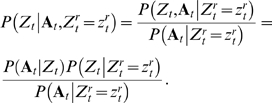
(10)Recall that in the coupled-pruning algorithm, we can readily compute all the three conditional probability terms in the above equation.

Performing posterior inference allows us to make motif predictions in two ways. A simple way is look at blocks in the alignment at which the posterior inference produces ones, and predict those to be motifs. Alternatively, we can also use the inferred state of the alignment block together with the inferred ancestral state to compute a probability score (as a heuristic) based on the functional annotation tree. The score for the block is the sum of probabilities of each block element being one.

### Tree Estimation

Given blocks of aligned substrings {**A** *_t_*} containing motif instances in at least one of the aligned taxa, in principle we can estimate both the *annotation tree T_f_* ≡ {*α*, *τ_f_*, *β_f_*} and the *motif trees T_m_* ≡ {*θ*, *τ_m_*, *β_m_*, *λ_m_*} based on a maximum likelihood principle. But since in our case most training CRM sequences do not have enough motif data to warrant correct estimation of the motif and function tree, we use the topology and branch lengths of a tree estimated by fastDNAml [36] from the entire CRM sequence alignment (containing both motif and background) as the common basis to build the *T_f_* and *T_m_*. Specifically, fastDNAml estimates a maximum likelihood tree under the F84 model from the entire CRM alignment; we then scale the branch lengths of this tree to get the sets of branch lengths for *T_f_* and *T_m_* by doing a simple linear search (see below) of the scaling coefficient that maximize the likelihood of aligned motif sequences and aligned annotation sequences, under the *T_m_* and *T_f_* (scaled based on the coefficients) respectively.

For simplicity, we estimate the background tree *T_b_* ≡ {*θ*, *τ_b_*, *β_b_*, *λ_b_*} separately from only aligned background sequences that are completely orthologous (i.e., containing no motifs in any taxon).

For both motifs and background phylogenies, the Felsenstein rate parameter *μ* for the corresponding nucleotide substitution models must also be estimated from the training data. More technically, note that for *T_m_* the scaling coefficient *β* and the rate parameter *μ* form a product in the expression of the substitution probability (see Equation 3) and are not identifiable independently. Thus we only need to estimate the compound rate parameter *μ′* = *μβ*. Ideally, the optimal value of the *μ′* should be obtained by performing a gradient descent on the likelihood under the corresponding phylogeny with respect to *μ′*. However, due to the phylogenetic tree probability terms involved in the likelihood computation, there is no closed form expression for the gradient that can be evaluated for a specific value of the compound rate parameter to determine the direction to choose for optimization. Therefore, to find an approximation to the optimal value of *μ′*, we perform a simple linear search in the space of *μ′* as follows:

**for** 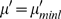 to 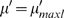 in steps of *δ* **do**
*L*(*μ′*) = Training motif likelihood under motif phylogeny *T* with compound Felsenstein rate *μ′*
**end for**
Choose *μ* that gives maximum likelihood: 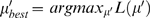

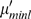
 and 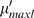 are lower and upper bounds respectively on the space of *μ′* that is searched, and are heuristically chosen based on observation. The step *δ* can be chosen to be as small as desired or is allowable, since having a smaller *δ* increases the number of values of *μ′* that must be tested and hence increases computation, but gives a more accurate optimum.

### Estimation of HMM Parameters

**Figure 13:**
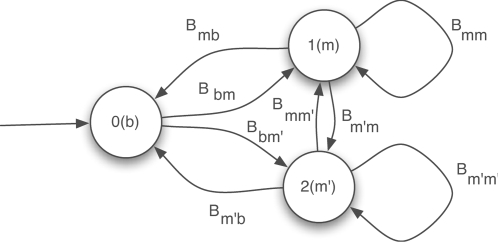
A 3-state HMM for a single motif.

For prediction of motifs and non-motifs on test sequences, we use an HMM to find the highest probability state (i.e., motif or background) at each site. The parameters for the HMM are the initial probability vector *π* and the transition probability matrix **B**. In the simplest scenario, when binding sites are to be searched for one TF at a time, the basic HMM only needs to model transitions among three different functional states: the background state (indicated by 0), the forward-motif state (indicated by 1) which indicates that the current site is the start of a motif on the forward DNA strand, and a reverse-motif state (indicated by 2) which indicates that the current site is the end of a motif on the reverse-complementary strand. Figure 13 shows the HMM corresponding to this scenario.

The initial probabilities are fixed by assuming that the HMM always starts in the background state. Thus, *π*_0_ = 1 and *π*_0_ = *π*_0_ = 0. For the transition matrix, we use the maximum likelihood estimator for transition from state *i* to state *j* (which has probability *B_i,j_*), this is given by the count of the number of such events in the training data divided by the total number of sites in state *i*. We follow the no-strand-bias assumption, and allow equal transition probabilities from the background state to both the forward-motif and reverse-motif states. Also, in the case where we do not have annotated training alignments, we can use the Baum-Welch algorithm for unsupervised estimation of the transition probability matrix.

### Comparison of CSMET to Available Software

We compare CSMET with four other programs—PhyloGibbs, EMnEM, PhyloHMM and Stubb. PhyloGibbs is chosen as it is presently a state of the art in multi-species motif detection [9] and it handles motif turnover. PhyloGibbs is an unsupervised algorithm for *de novo* motif detection, and it can also optionally run in supervised mode given PWM for motif search. For a fair comparison, we run PhyloGibbs by specifying the motif PWM based on a maximum likelihood estimation from training data. We run PhyloGibbs with the default set of parameters. We approximately specify the number of motifs expected to be seen, as needed by PhyloGibbs, since the actual number of conserved motifs can vary a lot in both our simulated data and in real biological data.

EMnEM is chosen as it is another popular multi-species motif detection algorithm based on a different phylogenetic model that does not handle motif turnover and evolutionary-rate autocorrelation. EMnEM performs *de novo* motif detection, but also has a supervised motif search mode, which we choose to operate on. Again, we also approximately specify the number of motifs expected to be seen, and run EMnEM with the default set of parameters.

PhyloHMM is chosen since it is a direct analog of CSMET, which assumes functional homogeniety across aligned sites. Available PhyloHMM-based tools are implemented for detecting genes [5] and conserved regions [23],[24], but no PhyloHMM implementations were available for motif finding. Hence, we implemented our own in-house PhyloHMM for the purpose of supervised motif detection.

Finally, Stubb is chosen as a representative single-species HMM based motif finder to investigate the advantage of comparative-genomic motif detection over traditional approaches that treat each species independently. Stubb can be run both as a single species or as an aligned two species model. Since we are interested in comparing our performance with single species motif detector, we use the single species mode. Also, it might not always be apparent as to which two species to compare in order to get the most meaningful contrast for separating functional sites and non-functional sites. Stubb was run individually on all the aligned sequences, with all the results collated for analysis.

### Data Processing and Experimental Setup

The synthetic CRMs where true TFBS annotations are known for evaluating CSMET are generated according to the scheme outlined in Figure 4. Given each 1500 bp simulated multiple alignment, we use 1000 bp for training, and the remaining 500 for testing the performance of the trained models. Details of the simulation procedure and the experimental setup are available in the Supplemental Materials.

Our biological dataset was created based on the motif database in [38],[39], from which we chose to predict TFBS of TF which have at least 10 or more biologically validated training instances. The five TFs which met this requirement were Bicoid, Caudal, Kruppel, Knirps and Hunchback motifs. Motif finding was performed on 14 CRMs listed in Table 1 which contained instances for these 5 binding sites. The multiple sequence alignment corresponding to the CRMs were obtained by using the UCSC Genome Browser pre-compiled alignments [40]. The sequence corresponding to *willistoni* was left out due to poor alignment quality and missing contigs. Flanking regions of 1000 bp on each side of the CRMs were also analyzed. For each CRM alignment, we use the motifs identified in *melanogaster* as references to mark all alignment blocks that contain at least one instance of motifs among the 11 taxa to be analyzed. As a result our benchmark is biased toward *melanogaster*, because annotations in other taxa are not available to mark motifs that are present in other *Drosophila* taxa but not in *melanogaster*. The *melanogaster* CRMs contain both biologically validated motifs and computationally identified but plausible motifs, as documented in [38],[39].

To train the CSMET, we manually annotated the functional states (i.e., *Z_t_*) across all taxa in all alignment blocks (i.e., **A** *_t_*) containing the *melanogaster* motif. We employ a 1 versus *K*−1 cross-validation scheme for testing on each motif type, where *K* is the total number of CRMs where a motif type is present. Specifically, for each motif type we trained all programs on *K*−1 out of the *K* CRMs hosting the motif, and tested on the remaining one, and we iterated this until all *K* CRMs had been tested. Recall that the test accuracy is assessed only for reported motifs in *melanogaster*, but not on those manually annotated ones in other taxa.

To avoid overfitting the motif and functional phylogenies of CSMET under limited training data, for all our experiments, we used a single phylogenetic tree estimated from the entire training sequence alignment dataset as the un-scaled version of the motif and functional trees. We assumed that the *T_f_*'s of every type of motif share the same topology and branch lengths, but different equilibriums. Thus, *T_f_* can be fitted from a concatenation of motif-instance alignments of all types of motifs. For the motif sequence phylogenies, we enforced the trees at every site in the same motif have the same topology, branch length, and the Felsenstein total substitution rate, but different equilibriums. A second tree was estimated on background sites only, and was used as the background phylogeny.

To handle real data which contains gaps and other complexities, it is necessary to change some settings of the competing software from their defaults to ensure proper behavior. EMnEM was run with default parameters, but with the threshold set to 0.999 to reduce false positives; as for the suggested threshold of 0.5, virtually every location was being classified as a motif. PhyloGibbs was run with default parameters, but for handling gaps, the modes of using the full alignment, as well as using partial alignments were tried, and the pre-estimated phylogeny on all species for the entire sequence was given to it. PhyloHMM was run naively using posterior decoding. Stubb was run with default settings with a slightly reduced threshold of 6.0. At the suggested threshold of 10.0 for a window size of 500, Stubb predicts no true positives.

### Evaluation

We base our evaluation of every program on three commonly used evaluation metrics - precision, recall and the F1 score (i.e., the harmonic mean) based on precision and recall [37]. The precision is defined as the ratio of number of true predicted positives over number of all predicted instances; and recall is defined as the number of true predicted positives to the number of all positives in the gold-standard annotation. (By this choice of evaluation score we avoided trivial specificity measure due to very large number of both predicted and true negatives.) We also allow a little leeway in the prediction of the motif location—a predicted hit falling within a tolerance window of size 5bp on either side of the actual starting location of the motif is also counted as a correct hit. When an algorithm fails to make any predictions, both precision and recall are taken to be zero. F1 score in such cases is also taken to be zero. For simulation-based evaluation, since the ground-truth of motif locations is known in all taxa, the numbers of true and false predictions are counted over motif instances in all taxa. For each experiment, we report summary statistics of performance scores over all 50 alignments for each algorithm.

## Supporting Information

Text S1 Supplementary material for methods.
(1.40 MB PDF)
