# Supplementary material for: CSMET: Comparative Genomic Motif Detection via Multi-Resolution Phylogenetic Shadowing
Source: PLoS Comput Biol. 2008 Jun 6;4(6):e1000090. doi: 10.1371/journal.pcbi.1000090 (PMC2396503; doi:10.1371/journal.pcbi.1000090)
Supplement: Text S1 — Supplementary material for methods. (1.40 MB PDF) [file pcbi.1000090.s001.pdf]

Supplementary Material for CSMET: Comparative Genomic Motif Detection via Multi-Resolution Phylogenetic Shadowing

Pradipta Ray *, Suyash Shringarpure *, Mladen Kolar, and Eric P. Xing†

School of Computer Science,
Carnegie Mellon University.

February 22, 2008

1 Materials and Methods

1.1 The Molecular and Functional Substitution Model

We use the Felsenstein 1984 model (F84) (1), which is similar to the Hasegawa - Kishino - Yano's 1985 model (HKY85) (2) and widely used in the phylogenetic inference and footprinting literature (1; 4), for nucleotide substitution in our motif and background phylogeny. Formally, F84 is a five-parameter model, based on a stationary distribution $\pi \equiv [\pi_A, \pi_T, \pi_G, \pi_C]'$ (which constitutes three free parameters as the equilibrium frequencies sum to 1) and the additional parameters κ and ι which impose the transition/transversion bias. Using concise notation for the purine frequency $\pi_R = \pi_A + \pi_G$ and pyrimidine frequency $\pi_Y = \pi_T + \pi_C$, the instantaneous rate matrix can be written as:

$$\mathbf{Q}_N = \begin{pmatrix} * & (1+\kappa/\pi_Y)\iota\pi_C & \iota\pi_A & \iota\pi_G \\ (1+\kappa/\pi_Y)\iota\pi_T & * & \iota\pi_A & \iota\pi_G \\ \iota\pi_T & \iota\pi_C & * & (1+\kappa/\pi_R)\iota\pi_G \\ \iota\pi_T & \iota\pi_C & (1+\kappa/\pi_R)\iota\pi_A & * \end{pmatrix} \quad (1)$$

Since rows of the instantaneous rate matrix must sum to zero, the starred elements of the matrix are determined from the other 3 elements of the row, and not shown for clarity. According to the continuous-time Markov process theory, the corresponding nucleotide-substitution probability matrix over a period of time t is given by $P_N(t) = e^{Q_N t}$. To apply this model to a motif or a background phylogeny, we set the stationary distribution π to be the empirical nucleotide-frequency in the corresponding sequence entity that the phylogeny is defined on (e.g., for phylogeny $T_m^{(l)}$ defined on site l of a motif, we let $\pi \equiv \theta_l$, the l-th column of the PWM of the motif), and the nucleotide-substitution probability from an internal node c to its descendant c' along a tree branch of length b can be expressed as follows:

$$P_N(V_{c'} = j | V_c = i, \beta) = e^{-(\kappa+\iota)\beta}\delta_{ij} + e^{-\iota\beta}(1 - e^{-\kappa\beta})\Big(\frac{\pi_j}{\sum_h(\pi_h\epsilon_{jh})}\Big)\epsilon_{ij} + (1 - e^{-\iota\beta})\pi_j, \quad (2)$$

*P.R. and S.S. contributed equally to the paper, and should be recognized as co-first authors.
†To whom the correspondence should be addressed.

where i and j denote nucleotides, δ_{ij} represents the Kronecker delta function, and ϵ_{ij} is a function similar to the Kronecker delta function which is 1 if i and j are both pyrimidines or both purines, but 0 otherwise. The summation in the denominator concisely computes π_R or π_Y.

A less concise, but more intuitive parameterization involves the overall substitution rate per site μ and the transition/transversion ratio ρ, which can be easily estimated or specified. We can compute the transition matrix P_N from μ and ρ using Eq. (2) based on the following relationship between (κ, ι) and (μ, ρ):

$$\kappa = \frac{2\pi_R\pi_T\rho - (2\pi_A\pi_G + 2\pi_C\pi_T)}{(2\pi_A\pi_G/\pi_R + 2\pi_C\pi_T/\pi_Y)} \frac{\mu}{1+\rho}, \qquad \iota = \frac{1}{2\pi_R\pi_Y} \frac{\mu}{1+\rho}.$$

To model functional turnover of aligned substrings along functional phylogeny T_f, we additionally define a substitution process over two characters (0 and 1) corresponding to presence or absence of functionality. Now we use the Jukes-Cantor 1969 model (JC69) (3) for functional turnover due to its simplicity and straightforward adaptability to an alphabet of size 2. The JC69 model is a single parameter model, using an instantaneous substitution rate μ which is confounded with the time variable. The instantaneous rate matrix under JC 69 is:

$$\mathbf{Q}_F = \begin{pmatrix} -\mu & \mu \\ \mu & -\mu \end{pmatrix}. \tag{3}$$

And the transition probability along a tree branch of length β (which now represents the product of substitution rate μ and evolution time t, which are not identifiable independently,) is defined by:

$$\mathbf{P}_F = \begin{pmatrix} \frac{1}{2} + \frac{1}{2}e^{-2\beta} & \frac{1}{2} - \frac{1}{2}e^{-2\beta} \\ \frac{1}{2} - \frac{1}{2}e^{-2\beta} & \frac{1}{2} + \frac{1}{2}e^{-2\beta} \end{pmatrix}. \tag{4}$$

From Eqs. (2) and (4), we can see that the likelihood of aligned nucleotides and functional states can be expressed as a function of the evolutionary parameters, based on which a maximum likelihood estimation of these parameters can be obtained from training data. Figure 1 outlines the procedure of maximum likelihood training of CSMET.

1.2 Multi-specific CRM simulation and experimental setup

The synthetic CRMs where true TFBS annotations are known for evaluating CSMET are generated as follows. First, the simulator stochastically samples the evolutionary trees of motif, background, and functional-annotation, T_m, T_b and T_f, from the prior distributions (recall that each tree is a three-tuple including the stationary distribution, the tree topology, and the branch lengths). The Felsenstein transition/transversion coefficient can in principle be also sampled, but for simplicity and biological validity we pre-specify it to be 2. Then it simulates motif instances, background sequences, and functionality states (that determine motif turnover) in different taxa from their respective evolutionary trees under certain substitution rates. It can also simulate motifs with changing substitution rates according to a scheduling along a sequence, or in random order. Then it uses the global HMM to generate positional organization of the motifs and backgrounds in the CRM. Finally these building blocks are put together to synthesize an artificial CRM. This simulator can be used to simulate realistic multi-specific CRMs resulting from various nontrivial evolutionary dynamics. It is useful in its own right for consistency/robustness analysis of motif evolution models and performance evaluation of comparative genomic motif-finding programs.

We performed three sets of simulation experiments based on simulated datasets. In each case, we generate a data set of CRM alignments from the simulator that is simulating a pre-specified coupled functional and molecular evolution processes unknown to the programs used in the test phase. Each data set contains 50 simulated alignments, each of which is 1500 basepairs in length and includes 10 taxa whose divergence is controlled by the topologies and the branch lengths of the functional and molecular phylogenies being used. Each alignment contains instances of a single type of motif, whose length is set to be 8-bp. The parameters of the generative model used for the simulations are chosen to be representative of such parameters estimated from real biological data.

The density of motif instances is subject to a systematic adjustment for each data set over a wide range to generate problems of different degrees of difficulty.

The experiment for evaluating performance of CSMET under varying TFBS turnover rates was performed by using a different annotation tree for each experimental point. An initial benchmark evolutionary tree was chosen with branch lengths and topology based on estimation from actual nucleotide alignments on 11

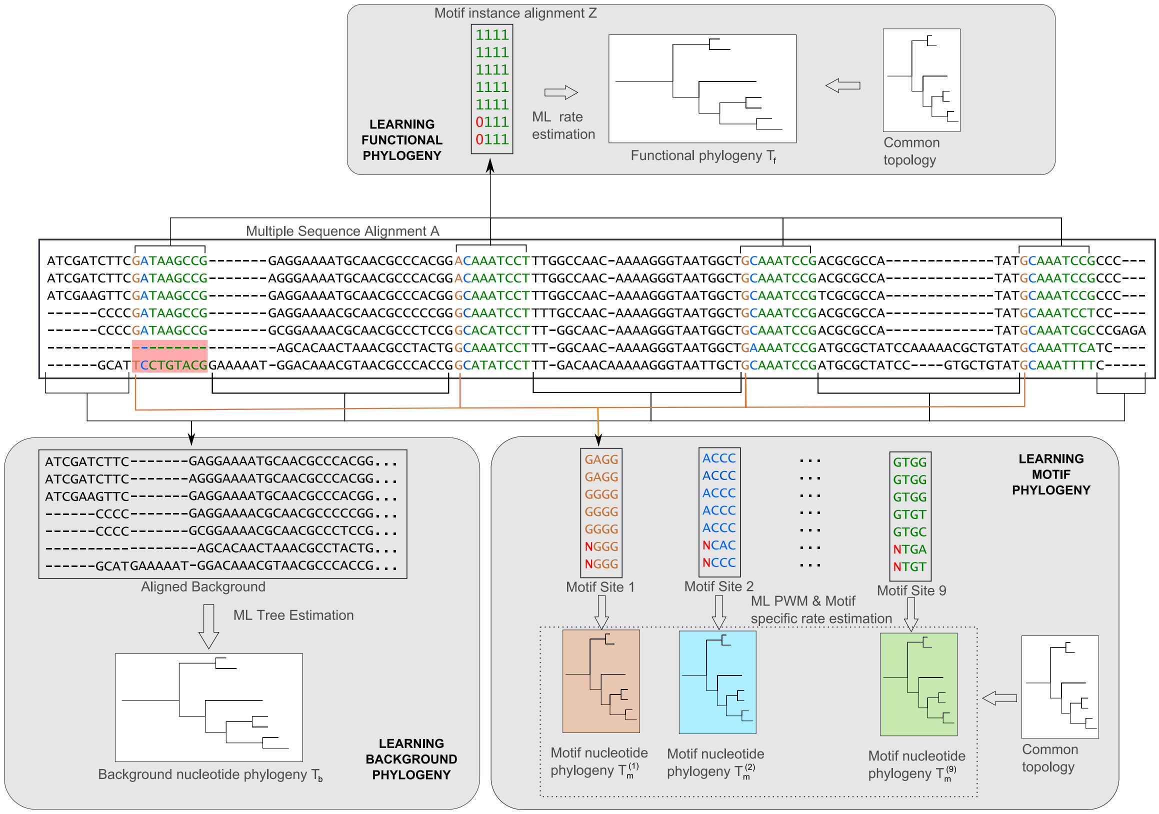

Figure 1: A schematic diagram of CSMET training. For the functional phylogeny, motif-instance alignments were generated by concatenating columns of indicators of motif presence/loss along the sequence alignment; and the scaling factor was fitted using the common topology. For the motif phylogeny, the nt-alignment of only each attendant site was generated by concatenating all columns of aligned nucleotides from that site and the corresponding multinomial estimated from them; the common topology was used for all sites. The motif specific mutation rate and scaling factor were estimated using the common topology from aligned nucleotides corresponding to all motif sites. For the background phylogeny, all segments of inter-motif sequences and flanking regions of CRMs were used.

aligned fly species. All parameters of the Jukes Cantor model based evolutionary tree were kept fixed across experimental data points, except for the fact that the branch lengths were scaled by a constant factor at each data point with respect to the initially chosen tree. The scaling factors correspondingly used for the data points were respectively: 1.50, 2.00, 2.50, 3.00, and 3.50. With increasing branch lengths, the amount of turnover per site in the simulated data increases - for a scaling factor tending to infinity the turnover model becomes random and approximates 50% For our data points, the estimated turnover rates corresponding to the chosen scaling factors were : 25%, 30%, 32%, 34% and 36%.

The simulated sequences with non-uniform TFBS turnover rates were generated by allowing the annotation tree scaling factor to vary for each motif block inside every simulated sequence. The scaling factor for each instance of a generated motif was equiprobably picked from the values of 1.00, 1.50, 2.00 and 2.50 . The corresponding turnover rates were 20%, 25%, 30% and 32%.

Given each 1500bp multiple alignment, we use 1000 bp for training, and the remaining 500 for testing the performance of the trained models. We base our evaluation of every program on three commonly used evaluation metrics - precision, recall and the F1 score (i.e., the harmonic mean) based on precision and recall (5).

References

[1] J. Felsenstein and G. A. Churchill. A Hidden Markov Model approach to variation among sites in rate of evolution. *Mol Biol Evol*, 13(1):93–104, 1996.

[2] M. Hasegawa, H. Kishino, and T. Yano. Dating of the human-ape splitting by a molecular clock of mitochondrial DNA. *J Mol Evol*, 22(2):160–74, 1985.

[3] T. H. Jukes and C. R. Cantor. Evolution of protein molecules. In H. N. Munro, editor, *Mammalian Protein Metabolism*, pages 21–32. Academic Press, New York, 1969.

[4] J. D. McAuliffe, L. Pachter, and M. Jordan. Multiple-sequence functional annotation and the generalized hidden markov phylogeny. *Bioinformatics*, 20:1850–1860, 2004.

[5] M. Tompa, N. Li, T. L. Bailey, G. M. Church, B. D. Moor, E. Eskin, A. V. Favorov, M. C. Frith, Y. Fu, W. J. Kent, V. J. Makeev, A. A. Mironov, W. S. Noble, G. Pavesi, G. Pesole, M. Regnier, N. Simonis, S. Sinha, G. Thijs, J. van Helden, M. Vandenbogaert, Z. Weng, C. Workman, C. Ye, and Z. Zhu. Assessing computational tools for the discovery of transcription factor binding sites. *Nat Biotechnol*, 23(1):137–44, 2005.
